# A high-throughput visual screening method for *p*-hydroxybenzoate hydroxylase to increase phenolic compounds biosynthesis

**DOI:** 10.1186/s13068-022-02142-w

**Published:** 2022-05-02

**Authors:** Zhenya Chen, Tongtong Chen, Shengzhu Yu, Yi-Xin Huo

**Affiliations:** Key Laboratory of Molecular Medicine and Biotherapy, School of Life Science, Beijing Institute of Technology, No. 5 South Zhongguancun Street, Haidian District, Beijing, 100081 China

**Keywords:** *p*-Hydroxybenzoate hydroxylase, Screening, Hydroxylation, Gallic acid, Pyrogallol, Biosynthesis

## Abstract

**Background:**

Gallic acid (GA) and pyrogallol are phenolic hydroxyl compounds and have diverse biological activities. Microbial-based biosynthesis, as an ecofriendly method, has been used for GA and pyrogallol production. In GA and pyrogallol biosynthetic pathways, the low hydroxylation activity of *p*-hydroxybenzoate hydroxylase (PobA) towards 3,4-dihydroxybenzoic acid (3,4-DHBA) limited the high-level biosynthesis of GA and pyrogallol.

**Results:**

This work reported a high activity PobA mutant (Y385F/T294A/V349A PobA) towards 3,4-DHBA. This mutant was screened out from a PobA random mutagenesis library through a novel naked eye visual screening method. In vitro enzyme assay showed this mutant has a *k*_cat_/*K*_m_ of 0.059 μM^−1^ s^−1^ towards 3,4-DHBA, which was 4.92-fold higher than the reported mutant (Y385F/T294A PobA). Molecular docking simulation provided the possible catalytic mechanism explanation of the high activity mutant. Expression of this mutant in *E. coli* BW25113 (Fʹ) can generate 840 ± 23 mg/L GA from 1000 mg/L 3,4-DHBA. After that, this mutant was assembled into a de novo GA biosynthetic pathway. Subsequently, this pathway was introduced into a 3,4-DHBA-producing strain (*E. coli* BW25113 (Fʹ)Δ*aroE*) to achieve 301 ± 15 mg/L GA production from simple carbon sources. Similarly, assembling this mutant into a de novo pyrogallol biosynthetic pathway enabled 129 ± 15 mg/L pyrogallol production.

**Conclusions:**

This work established an efficient screening method and generated a high activity PobA mutant. Assembling this mutant into de novo GA and pyrogallol biosynthetic pathways achieved the production of these two compounds from glucose. Besides, this mutant has great potential for the production of GA or pyrogallol derivatives. The screening method could be used for other GA biosynthesis-related enzymes.

**Supplementary Information:**

The online version contains supplementary material available at 10.1186/s13068-022-02142-w.

## Background

Gallic acid (GA), a natural phenolic acid of plants, has diverse biological activities such as antioxidant, antibacterial and anti-inflammatory activities [1–7]. Pyrogallol, a decarboxylated product of GA, exhibits similar biological activities as GA [8, 9]. Besides, pyrogallol has broad-spectrum antiseptic activity [10, 11]. According to the properties of GA and pyrogallol, these two compounds have been widely applied in food, pharmaceutical and cosmetic industries [12, 13]. For instance, GA can recover the antioxidase activity in the liver, brain and kidney of senescence accelerated mice [14]. Pyrogallol was a crucial precursor for synthesizing antianginal drug trimethoxybenzidine [15].

Traditional method of GA production mainly relies on the hydrolysis of tannins through acids or bases [16]. In addition, GA can be alternatively produced by fermentation of tannins using the strain with active tannase [17–19]. The main approach for pyrogallol production was decarboxylation of GA via toxic chemicals under harsh conditions. Besides, enzymatic decarboxylation which was induced by 3,4-dihydroxybenzoic acid decarboxylase (PDC), was also utilized to produce pyrogallol [20]. The PDC-induced decarboxylation reaction required an anaerobic environment. Besides, the purification of PDC is complicated and time-consuming. The abovementioned methods for GA and pyrogallol production also suffered from other drawbacks, such as the limited amount of raw materials, harsh reaction conditions, environment pollution and low yield [21].

In recent years, microbial-based biosynthesis has been attempted to produce many value-added compounds, including GA and pyrogallol [22, 23], from simple carbon sources [24–29]. GA and pyrogallol biosynthetic pathways generally extended from 4-hydroxybenzoic acid (4-HBA), an *Escherichia coli* (*E. coli*) endogenous compound generated from the shikimate pathway [22]. 4-HBA can be catalyzed to form GA through a two-step reaction, which included hydroxylation of 4-HBA into 3,4-dihydroxybenzoic acid (3,4-DHBA) and hydroxylation of 3,4-DHBA into GA. This two-step reaction was generally activated by *p*-hydroxybenzoate hydroxylase (PobA). For pyrogallol biosynthesis, PDC was generally introduced into GA-producing strain to convert GA into pyrogallol [23]. Native PobA from *Pseudomonas fluorescens* (*P. fluorescens*) displays high hydroxylase activity towards 4-HBA, but very weak or negligible activity towards 3,4-DHBA [30]. To avoid using PobA, Wang et al. designed an alternative pathway to achieve 1035.75 mg/L pyrogallol production [31]. To address the weak activity of PobA towards 3,4-DHBA, previous studies mutated the tyrosine at 385th position of PobA into phenylalanine, which led PobA to hydroxylate 3,4-DHBA into GA [30, 32]. Based on that, Kambourakis et al. utilized this mutant to engineer a GA-producing strain and this strain produced 20 g/L GA in a fermenter [20]. Further, Chen et al. rationally designed a higher activity mutant (Y385F/T294A PobA) towards 3,4-DHBA [22]. After that, Moriwaki et al. acquired the tertiary structure of Y385F PobA and rationally designed a high activity mutant (L199V/Y385F PobA) [33]. Maxel et al. established a growth-based screening method and utilized this method to screen two high activity mutants (L199R/T294C/Y385M PobA and V47I/L199N/T294A/Y385I PobA) [34].

Though PobA mutants with the ability of hydroxylating 3,4-DHBA have been obtained, the hydroxylation activity still cannot satisfy the demand of high-level GA and pyrogallol production. Specifically, the low activity of PobA towards 3,4-DHBA can lead carbon source to flow into the byproduct catechol biosynthetic pathway. Therefore, PobA mutant with higher activity urgently needs to be investigated. Rational design of PobA mutants generally required to deeply understand the catalytic mechanism of PobA. In some cases, the mutants generated from rational design did not have the expected high activity [35]. Random mutagenesis combined with an effective and convenient screening method has high probability to obtain ideal PobA mutants. In this study, we established an efficient and simple method for screening high activity PobA mutants. This method depended on the instability of GA in alkaline conditions and the generated degradation products could react with each other to form a phenolic mixture [36, 37]. Specifically, the mixture has a green color visible to naked eyes and the maximum absorption wavelength was at 640 nm. Based on that, we adopted this method to screen out a high activity mutant (Y385F/T294A/V349A PobA) from PobA random mutagenesis library. This mutant has higher in vitro catalytic efficiency and in vivo conversion ability than the reported mutant (Y385F/T294A PobA). After that, this mutant was demonstrated in engineered *E. coli* to produce GA or pyrogallol. Assembling Y385F/T294A/V349A PobA into de novo GA or pyrogallol biosynthetic pathway achieved the efficient biosynthesis of the corresponding products. This work constructed an efficient screening method and then used this method to screen out a high activity PobA mutant.

## Results and discussion

### Confirmation of GA performance in alkaline conditions

**Fig. 1:**
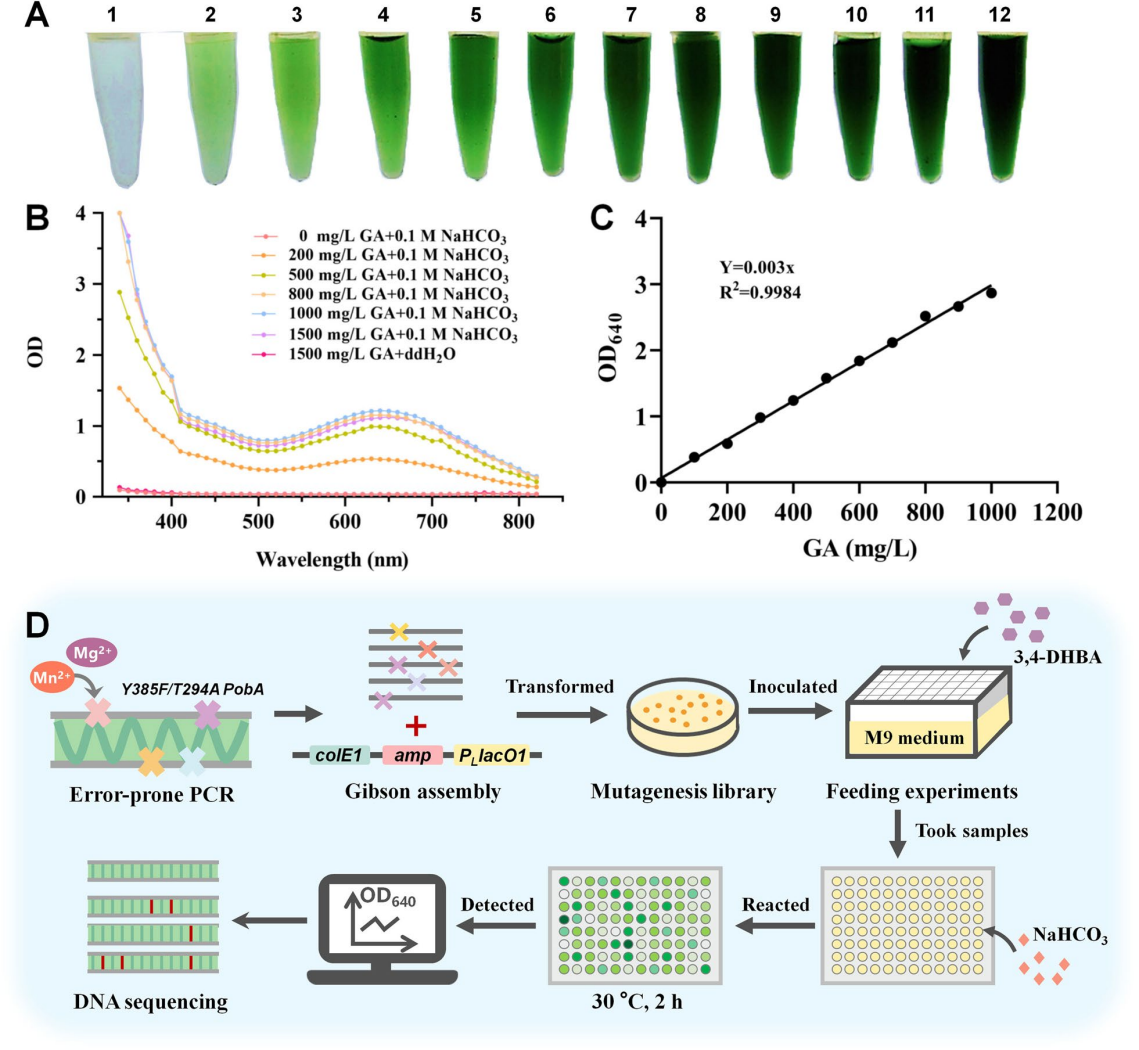
GA performance in alkaline conditions and the whole screening process of PobA mutants. **A** The reaction mixture of GA and NaHCO_3_. GA concentrations in tube 1–12 were 0, 0.1, 0.2, 0.3, 0.4, 0.5, 0.6, 0.7, 0.8, 0.9, 1.0, 1.5 g/L, respectively. NaHCO_3_ concentration in the tube was 1 M. **B** The full wavelength (340–820 nm) scan results of the mixture. **C** The linear relationship of GA concentration and the optical density at 640 nm. **D** The whole screening process for screening high activity PobA mutants

This study aimed to acquire the PobA mutants with high hydroxylation activity towards 3,4-DHBA. As a product GA is unstable in alkaline conditions and the degradation products can react with each other to form a phenolic mixture [36–40]. In this work, we firstly mixed GA and alkali NaHCO_3_. After 2 h, we found the mixture with a pH of 9.3 displayed green color visible to naked eyes (Fig. 1A). Moreover, the green color deepened as GA concentration increased. Besides, UHPLC and MS were used to analyze the mixture. Additional file 1: Fig. S1 shows 200 mg/L GA can be completely degraded in 2 h. MS results in Additional file 1: Fig. S2A show several new compounds were formed in the mixture. Based on the formula weights of the new compounds, we speculated one of the new compounds might be ellagic acid (Additional file 1: Fig. S2B), whose amount was highest in the mixture. The other compound might be 3,8-dihydroxy-2-(2-hydroxyethoxy)-7-(2,3,3-trihydroxypropoxy)chromeno[5,4,3-cde]chromene-5,10-dione (Additional file 1: Fig. S2C). Subsequently, the mixture was scanned at full wavelength (340–820 nm). Figure 1B shows the mixture has a maximum absorption wavelength of 640 nm. We then confirmed the relationship between OD_640_ value and GA concentration of the mixture. GA concentration exhibited linear relationship with OD_640_ value (Fig. 1C). These results demonstrate through adding NaHCO_3_, the change of GA concentration can be observed by naked eyes, and GA concentration can be confirmed through detection of OD_640_ value. These suggest addition of NaHCO_3_ in the stopped reaction of PobA hydroxylating 3,4-DHBA can be used as an efficient strategy to screen PobA mutants.

### Screening of PobA mutants and measurement of kinetic parameters

According to the performance of GA in NaHCO_3_ liquid, we designed a complete screening process for PobA mutants (Fig. 1D). Firstly, error-prone PCR was conducted on gene *Y385F/T294A pobA*, generating a PobA mutagenesis library. The single colonies of the library were pre-incubated into 96-deep-well plates containing LB medium, and the pre-inoculum was then transferred into another 96-deep-well plates containing M9Y medium with 1000 mg/L substrate 3,4-DHBA. After 12 h, the culture samples were taken. Then, the samples were centrifuged and the supernatant was put into 96-well plates which contained 0.1 M NaHCO_3_. After reaction for 2 h, the samples with darker green color than the control were picked out and then re-screened through detection of OD_640_ value. Based on that, a high activity mutant (Y385F/T294A/V349A PobA) was screened out from PobA mutagenesis library. Subsequently, this mutant was expressed and purified. SDS-PAGE in Additional file 1: Fig. S3 shows the purity of purified Y385F/T294A/V349A PobA was greater than 95%. We firstly used HPLC to detect the amount of GA after reaction for 2 min. However, because of the short reaction time the amount of GA was too low to be detected. If the reaction time was extended to increase the GA production, the initial reaction rate cannot be detected accurately. Considering the method of measuring NADPH oxidation at 340 nm was a commonly used method to calculate the kinetic parameters of NADPH-dependent oxidoreductases [22, 33, 34, 41, 42], we then employed this method to calculate the kinetic parameters of PobA mutants. The non-linear regression curves of PobA mutants towards 4-HBA and 3,4-DHBA through the Michaelis–Menten equation are shown in Additional file 1: Fig. S4. The *K*_m_ of Y385F/T294A/V349A PobA was 30.3 ± 10.4 μM towards 3,4-DHBA (Table 1), which was 4.22-fold lower than that of the reported mutant (Y385F/T294A PobA), suggesting that Y385F/T294A/V349A PobA has stronger affinity towards 3,4-DHBA when compared with Y385F/T294A PobA. Besides, Y385F/T294A/V349A PobA has a *k*_cat_/*K*_m_ of 0.059 μM^−1^ s^−1^ towards 3,4-DHBA, a 4.92-fold higher value when compared with that of Y385F/T294A PobA. The *k*_cat_/*K*_m_ of Y385F/T294A/V349A PobA towards 3,4-DHBA was close to that of other reported mutants [33, 34]. Besides, the *k*_cat_/*K*_m_ of Y385F/T294A/V349A PobA towards 4-HBA was 0.094 μM^−1^ s^−1^, which was 5.22-fold higher than that of Y385F/T294A PobA. These indicate Y385F/T294A/V349A PobA possesses higher catalytic efficiency towards 4-HBA or 3,4-DHBA than Y385F/T294A PobA towards 4-HBA or 3,4-DHBA.

**Table 1:** Kinetic parameters of PobA mutants towards 4-HBA and 3,4-DHBA

PobA mutants	4-HBA				3,4-DHBA			
	*V*_max_ (μM s^−1^)	*K*_m_ (μM)	*k*_cat_ (s^−1^)	*k*_cat_/*K*_m_ (μM^−1 ^s^−1^)	*V*_max_ (μM s^−1^)	*K*_m_ (μM)	*k*_cat_ (s^−1^)	*k*_cat_/*K*_m_ (μM^−1^ s^−1^)
Chen et.al								
Wild type	0.350 ± 0.04	34.7 ± 9.5	14.1 ± 1.5	0.41	–	–	–	–
Y385F	0.200 ± 0.001	19.6 ± 0.3	0.200 ± 0.010	0.01	0.390 ± 0.060	228 ± 54	0.390 ± 0.06	0.002
Y385F/T294A	0.450 ± 0.010	48.4 ± 3.0	0.900 ± 0.030	0.02	0.840 ± 0.090	157 ± 31	1.69 ± 0.18	0.012
This study								
Y385F/T294A	0.800 ± 0.02	89.9 ± 11.2	1.60 ± 0.05	0.018	0.790 ± 0.12	128 ± 52	1.59 ± 0.32	0.012
Y385F/T294A/V349A	0.680 ± 0.02	14.5 ± 2.5	1.36 ± 0.04	0.094	0.890 ± 0.08	30.3 ± 10.4	1.78 ± 0.16	0.059

### Molecular docking simulation of PobA mutants

**Fig. 2:**
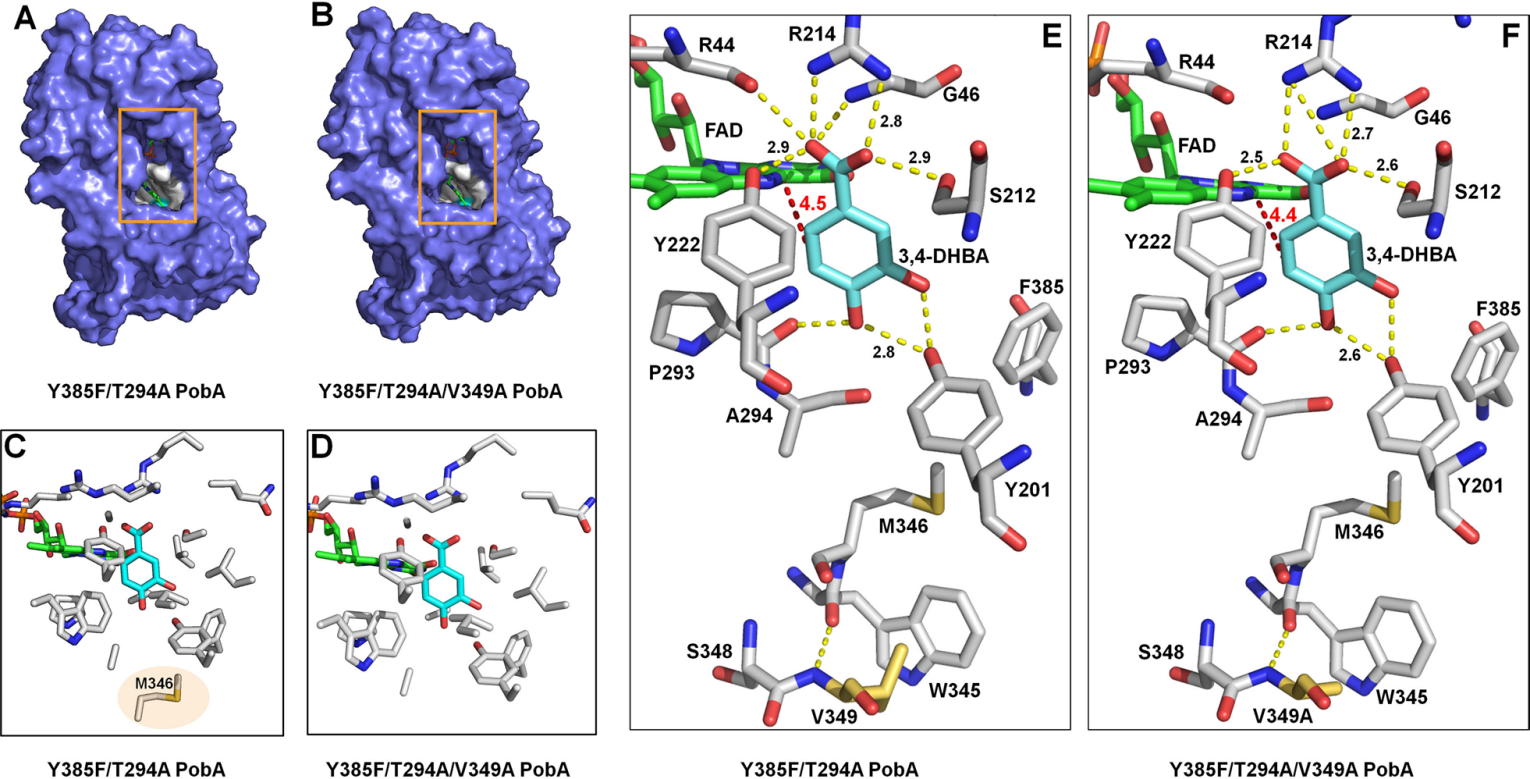
Modeling structures of PobA mutants with FAD and 3,4-DHBA. **A** Catalytic pocket of Y385F/T294A PobA with FAD and 3,4-DHBA complex. **B** Catalytic pocket of Y385F/T294A/V349A PobA with FAD and 3,4-DHBA complex. **C** Amino acid residues within the distance of 5 Å around 3,4-DHBA in Y385F/T294A PobA. **D** Amino acid residues within the distance of 5 Å around 3,4-DHBA in Y385F/T294A/V349A PobA. **E** Close view of the catalytic pocket of Y385F/T294A PobA with FAD and 3,4-DHBA complex. **F** Close view of the catalytic pocket of Y385F/T294A/V349A PobA with FAD and 3,4-DHBA complex. The hydrogen bonds were shown as dashed line

Subsequently, molecular docking simulation was conducted to provide mechanism explanation for the high activity of Y385F/T294A/V349A PobA. Y385F PobA (PDB code: 6JU1) was used as template for simulating Y385F/T294A PobA and Y385F/T294A/V349A PobA. After that, molecular docking of the mutants with substrate 3,4-DHBA and cofactor FAD were conducted to generate the corresponding complexes. As shown in Fig. 2A and B, Y385F/T294A PobA and Y385F/T294A/V349A PobA possess similar catalytic pocket. In the pocket, amino acid residues Y201 and P293 of PobA mutants, and 4-OH of 3,4-DHBA composed a hydrogen-bond loop, which was same as the complex of wild-type PobA with native substrate 4-HBA [22]. Besides, 3-OH of 3,4-DHBA formed hydrogen bonds with Y201 of PobA mutants. The unhydroxylated C5 atom of 3,4-DHBA pointed to the isoalloxazine ring of FAD, which was same as the orientation in the complex of Y385F with 3,4-DHBA and FAD (Additional file 1: Fig. S5) [33]. The distance between the C5 atom of 3,4-DHBA and the C4a atom of FAD was 4.5 Å in the complex of Y385F/T294A PobA with FAD and 3,4-DHBA (Fig. 2E), which was close to that in the complex of Y385F/T294A/V349A PobA with FAD and 3,4-DHBA (Fig. 2F). In the complex of wild-type PobA with 4-HBA and FAD, the unhydroxylated C3 atom of 4-HBA which was the hydroxylation site, also pointed to the isoalloxazine ring of FAD. Therefore, we speculated the catalytic mechanism of PobA mutants towards 3,4-DHBA was similar to that of wild-type PobA towards 4-HBA [33, 43, 44]. First, FAD cofactor in the complex is reduced by NADPH, which is responded to the binding of 3,4-DHBA to PobA mutants. Subsequently, the oxygen in environment oxidizes the reduced FAD to produce a hydroperoxide. The hydroperoxide then attacks the C–H bond at 5th position of 3,4-DHBA to generate a new hydroxyl group, forming product GA.

Besides, amino acid residues within the distance of 5 Å around 3,4-DHBA in Y385F/T294A PobA (Fig. 2C) and Y385F/T294A/V349A PobA (Fig. 2D) were compared. Compared to M346 of Y385F/T294A PobA, M346 of Y385F/T294A/V349A PobA (Fig. 2D) did not distribute within 5 Å, suggesting the mutation V349A induced the structural change of Y385F/T294A/V349A PobA. This change might reduce the obstruction of substrate 3,4-DHBA entry into catalytic pocket. Further, the hydrogen bonds between 3,4-DHBA and the amino acids in catalytic pockets were analyzed. Compared to the complex of Y385F/T294A PobA with FAD and 3,4-DHBA (Fig. 2E), the complex of Y385F/T294A/V349A PobA with FAD and 3,4-DHBA did not have the hydrogen bond between R44 and 1-COOH of 3,4-DHBA and the hydrogen bond between G46 and 1-COOH of 3,4-DHBA (Fig. 2F). In addition, a new hydrogen bond was formed between R214 of Y385F/T294A/V349A PobA and 1-COOH of 3,4-DHBA. Moreover, the hydrogen bond between Y222 of Y385F/T294A/V349A PobA and 1-COOH of 3,4-DHBA was shorter than the hydrogen bond between Y222 of Y385F/T294A PobA and 1-COOH of 3,4-DHBA. The hydrogen bond between S212 of Y385F/T294A/V349A PobA and 1-COOH was also shorter than the hydrogen bond between S212 of Y385F/T294A PobA and 1-COOH of 3,4-DHBA. These altered hydrogen bonds suggested that the position of the substrate 3,4-DHBA has changed, indicating V349A was able to endow the loop complex with more conformational flexibility. Compared to the solvent accessible surface area (SASA) of the binding pocket in Y385F/T294A PobA (1690.547 Å^2^), the SASA of the binding pocket in Y385F/T294A/V349A PobA was increased (1764.048 Å^2^). These suggested the binding pocket of Y385F/T294A/V349A PobA became bigger than that of Y385F/T294A PobA, which would provide more additional space for substrate 3,4-DHBA entering and then improve the binding of 3,4-DHBA to FAD and active amino acids of Y385F/T294A/V349A PobA, finally resulting in the high activity.

### Bioconversion of 3,4-DHBA into GA

**Fig. 3:**
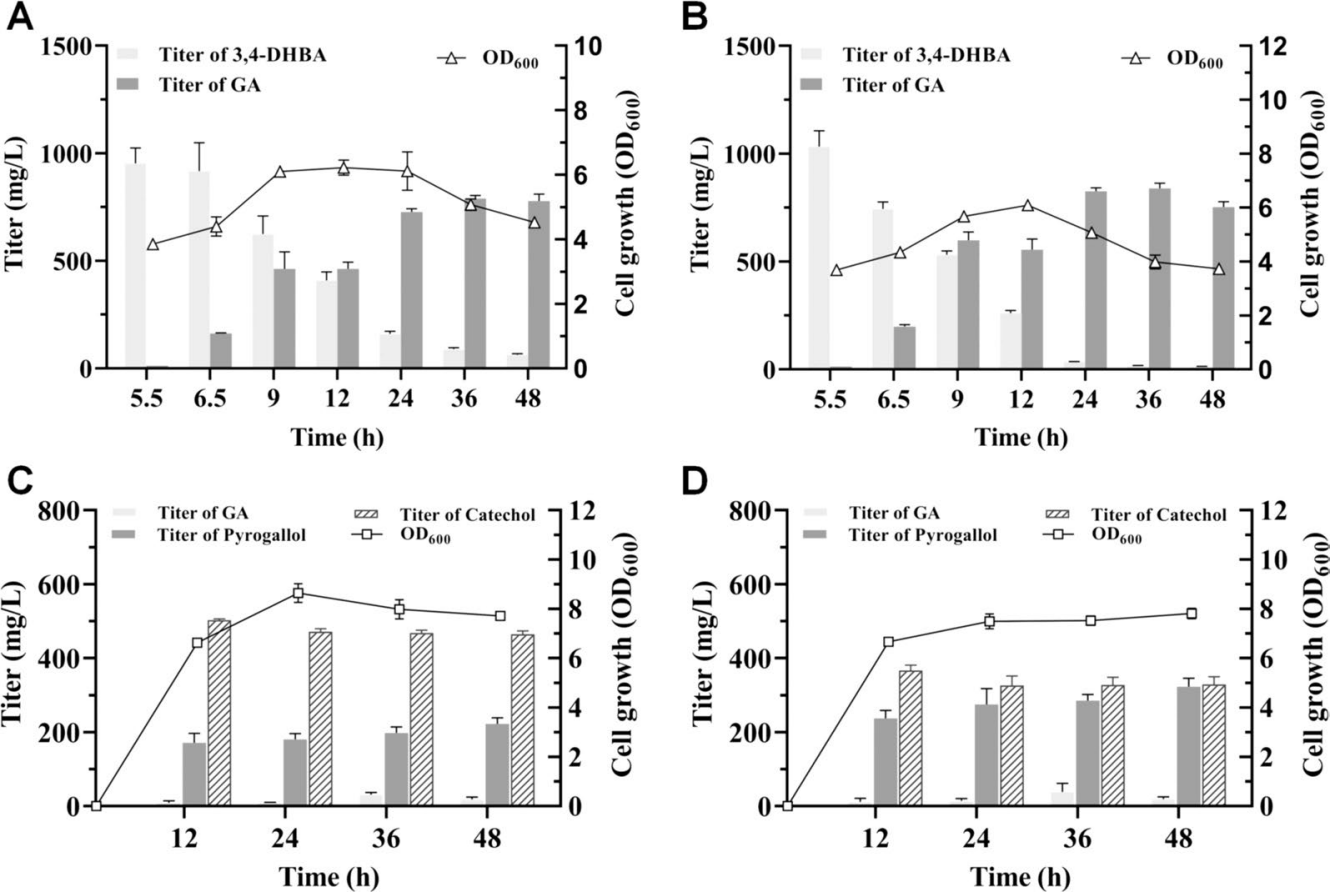
In vivo conversion of 3,4-DHBA into GA or pyrogallol. 3,4-DHBA with a concentration of 1000 mg/L was fed to the culture at 5.5 h. **A** Strain *E. coli* BW25113 (Fʹ) with pZE-PobA^**^ (CTT1) was used. **B** Strain *E. coli* BW25113 (Fʹ) with pZE-PobA^***^ (CTT2) was used. **C** Strain *E. coli* BW25113 (Fʹ) with pZE-PobA^**^ and pCS-PDC (CTT3) was used. **D** Strain *E. coli* BW25113 (Fʹ) with pZE-PobA^***^ and pCS-PDC (CTT4) was used

To test the in vivo conversion ability of PobA mutants towards 3,4-DHBA, Y385F/T294A PobA and Y385F/T294A/V349A PobA were individually expressed in *E. coli* BW25113 (Fʹ), generating strains CTT1 and CTT2, respectively. 1000 mg/L 3,4-DHBA was added to the culture at 5.5 h. As shown in Fig. 3A, CTT1 accumulated 163 ± 2 mg/L GA at 6.5 h, representing an initial in vivo conversion rate of 42.3 ± 0.5 mg/L/h/OD. Within 24 h, about 80% of 1000 mg/L 3,4-DHBA was consumed and 728 ± 15 mg/L GA was generated in the culture. In the next 24 h, the conversion ability of CTT1 decreased and few GA was produced. Similar to CTT1, 1000 mg/L 3,4-DHBA was also fed to CTT2 at 5.5 h. The results showed CTT2 produced 199 ± 8 mg/L GA in the culture at 6.5 h (Fig. 3B) and displayed an initial in vivo conversion rate of 54.1 ± 2.2 mg/L/h/OD, which was 1.28-fold higher than that of CTT1. Within 36 h, CTT2 almost completely consumed 1000 mg/L 3,4-DHBA and produced 840 ± 23 mg/L GA. Significantly, the titer of GA was 1.06-fold higher than that of CTT1 at 36 h. After this time point, GA titer was decreased due to the oxidation of GA. These results suggest that *E. coli* BW25113 (Fʹ) with Y385F/T294A/V349A PobA expression exhibits higher in vivo conversion ability towards 3,4-DHBA than *E. coli* BW25113 (Fʹ) with Y385F/T294A PobA expression.

### Bioconversion of 3,4-DHBA into pyrogallol

Further, in vivo conversion of 3,4-DHBA into pyrogallol were achieved by expressing PobA mutants and decarboxylase PDC in *E. coli* BW25113 (Fʹ). Plasmids pZE-PobA^**^ and pZE-PobA^***^ were individually introduced into *E. coli* BW25113 (Fʹ) harboring pCS-PDC, resulting in strains CTT3 and CTT4, respectively. 1000 mg/L 3,4-DHBA was fed to CTT3 or CTT4 at 5.5 h. As shown in Fig. 3C, CTT3 possessed a high growth rate in the first 12 h and has an OD_600_ value of 6.62 ± 0.05 at 12 h. Within 12 h, 9.17 ± 5.20 mg/L GA, 171 ± 26 mg/L pyrogallol and 503 ± 3 mg/L byproduct catechol accumulated in the culture. During the next 36 h, the titer of pyrogallol increased to 222 ± 16 mg/L at 48 h, while the titer of catechol decreased to 465 ± 9 mg/L. Though CTT3 can convert 3,4-DHBA into pyrogallol, large accumulation of byproduct catechol was accompanied.

As a comparison, CTT4 grew rapidly in the first 12 h and has an OD_600_ value of 6.66 ± 0.11 at 12 h (Fig. 3D). Meanwhile, pyrogallol with a titer of 237 ± 21 mg/L was detected in the culture, which was 1.39-fold higher than that of CTT3 at the same time point. In addition, the byproduct catechol has a titer of 367 ± 14 mg/L, a 1.37-fold lower value when compared with that of CTT3. Significantly, the titer of pyrogallol gradually increased to 323 ± 23 mg/L at 48 h, which was 1.45-fold higher than that of CTT3. These results indicate expressing PobA mutant and PDC in *E. coli* BW25113 (Fʹ) could achieve the in vivo conversion of 3,4-DHBA into pyrogallol. Moreover, Y385F/T294A/V349A PobA coupling with PDC represents higher in vivo ability of converting 3,4-DHBA into pyrogallol when compared with Y385F/T294A PobA coupling with PDC.

### Establishment of the biosynthetic pathway for 3,4-DHBA production

**Fig. 4:**
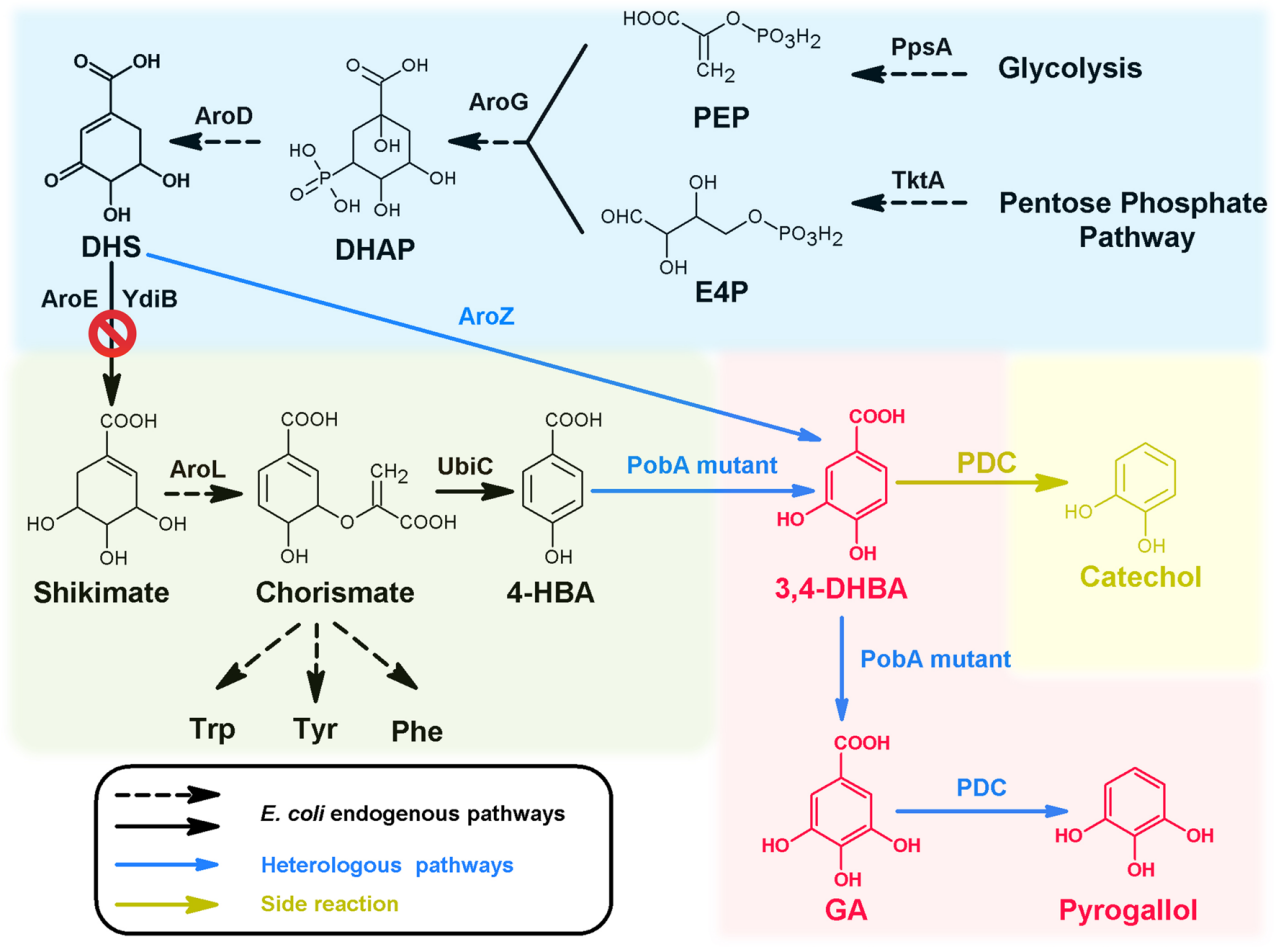
The de novo biosynthetic pathway of GA and pyrogallol. Black-colored arrows indicate the native pathways in *E. coli*; blue colored arrow indicates the heterologous steps; yellow-colored arrow indicates side-reaction step. PEP, phosphoenolpyruvate; E4P, *D*-erythrose 4-phosphate; DAHP, 3-deoxy-*D*-arabinoheptulosonate 7-phosphate; DHS 3-dehydroshikimate; 3,4-DHBA, 3,4-dihydroxybenzoic acid; GA, gallic acid; PpsA, phosphoenolpyruvate synthetase; TktA, transketolase; AroG, 2-dehydro-3-deoxyphosphoheptonate aldolase; AroD, 3-dehydroquinate dehydratase; AroE, shikimate dehydrogenase; YdiB, quinate/shikimate dehydrogenase; AroZ, 3-dehydroshikimate dehydratase; AroL, shikimate kinase II; UbiC, chorismate lyase; PobA mutant, *p*-hydroxybenzoate hydroxylase with mutations; PDC, 3,4-dihydroxybenzoic acid decarboxylase

**Fig. 5:**
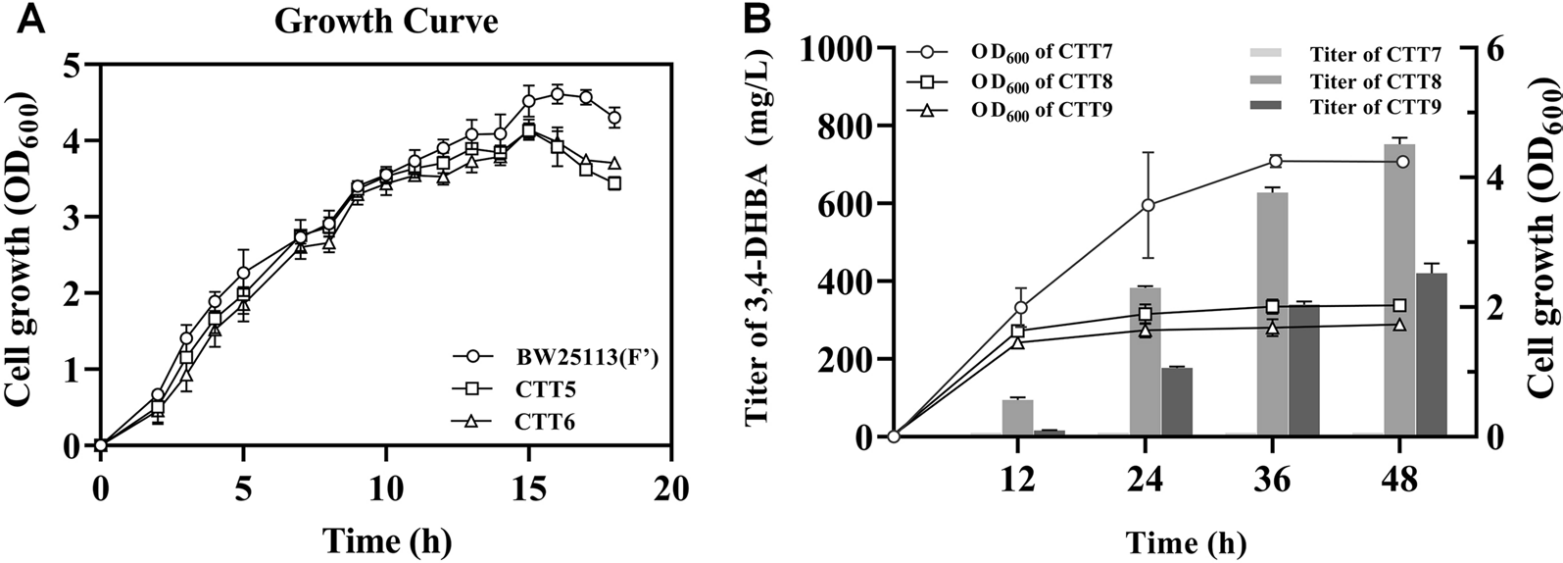
The cell growth and de novo production of 3,4-DHBA*.* **A** The growth curves of *E. coli* BW25113 (Fʹ), *E. coli* BW25113 (Fʹ)Δ*aroE* (CTT5) and *E. coli* BW25113 (Fʹ)Δ*aroE*Δ*ydiB* (CTT6) in LB medium. **B** 3,4-DHBA production of strains *E. coli* BW25113 (Fʹ) with pZE-AroZ (CTT7), CTT5 with pZE-AroZ (CTT8) and CTT6 with pZE-AroZ (CTT9) in M9Y medium

Construction of an efficient 3,4-DHBA biosynthetic pathway was significant for achieving the de novo production of GA and pyrogallol. In previous study, through expression of heterogenous PobA 3,4-DHBA can be produced from 4-HBA in *E. coli* [22]. For GA and pyrogallol production, heterogenous PobA required to catalyze two reactions, hydroxylating 4-HBA into 3,4-DHBA and hydroxylating 3,4-DHBA into GA (Fig. 4). Generally, the efficiency of two reactions induced by one kind of enzyme was lower than that of one reaction induced by one kind of enzyme. In this work, to avoid the issue of PobA-catalyzing two reactions and achieve efficient GA and pyrogallol production, *E. coli* BW25113 (Fʹ) was engineered to produce 3,4-DHBA from 3-dehydro-shikimate (DHS) (Fig. 4). Firstly, 4-HBA biosynthetic pathway in *E. coli* BW25113 (Fʹ) was blocked through knockout of gene *aroE* (strain CTT5) or knockout of genes *aroE* and *ydiB* (strain CTT6). AroE and YdiB are isoenzymes that can catalyze DHS to produce shikimate. Additional file 1: Fig. S6 shows CTT5 can grow in M9 medium, while CTT6 cannot grow in M9 medium because it cannot synthesize the essential amino acids phenylalanine, tyrosine and tryptophan. These results were consistent with the theoretical expectation. Subsequently, the growth curves of CTT5 and CTT6 were measured in LB medium. As shown in Fig. 5A, the OD_600_ values of CTT5 and CTT6 increased with the extension of culture time. At 15 h, CTT5 and CTT6 reached maximum OD_600_ values, 4.13 ± 0.10 and 4.15 ± 0.13, respectively. After this time point, the growth of CTT5 and CTT6 stopped. As a comparison, the original strain *E. coli* BW25113 (Fʹ) has a maximum OD_600_ value of 4.61 ± 0.13 at 16 h, which was close to that of CTT5 and CTT6 at 15 h. After 16 h, *E. coli* BW25113 (Fʹ) stopped growing. These results suggest knockout of *aroE* or knockout of *aroE* and *ydiB* in *E. coli* BW25113 (Fʹ) did not significantly affect the cell growth.

To achieve the de novo production of 3,4-DHBA, 3-dehydroshikimate dehydratase (AroZ) which can catalyze 3-dehydro-shikimate to produce 3,4-DHBA, was individually introduced into *E. coli* BW25113 (Fʹ), CTT5 and CTT6 to generate strains CTT7, CTT8 and CTT9, respectively. Figure 5B shows 3,4-DHBA titers of CTT8 and CTT9 continued to increase during the 48-h fermentation. The growth curves of CTT8 and CTT9 were similar. The OD_600_ values of CTT8 and CTT9 raised rapidly in first 12 h and have no significant improvement during the next 36 h. At 48 h, CTT8 produced 752 ± 17 mg/L 3,4-DHBA. Meanwhile, the OD_600_ value was 2.03 ± 0.06. For CTT9, 420 ± 26 mg/L 3,4-DHBA accumulated in the culture at 48 h, which was 1.79-fold lower than that of CTT8. These indicate the ability of strain CTT8 to produce 3,4-DHBA was higher than that of CTT9. As a comparison, CTT7 has negligible 3,4-DHBA accumulation throughout the 48-h fermentation, suggesting without knockout of *aroE* or *ydiB* *E. coli* BW25113 (Fʹ) could not synthesize 3,4-DHBA in large amount. These results suggest the engineered *E. coli* BW25113 (Fʹ) (CTT8 or CTT9) has ability to de novo produce 3,4-DHBA and can be used as host for de novo GA and pyrogallol production.

### De novo production of GA

**Fig. 6:**
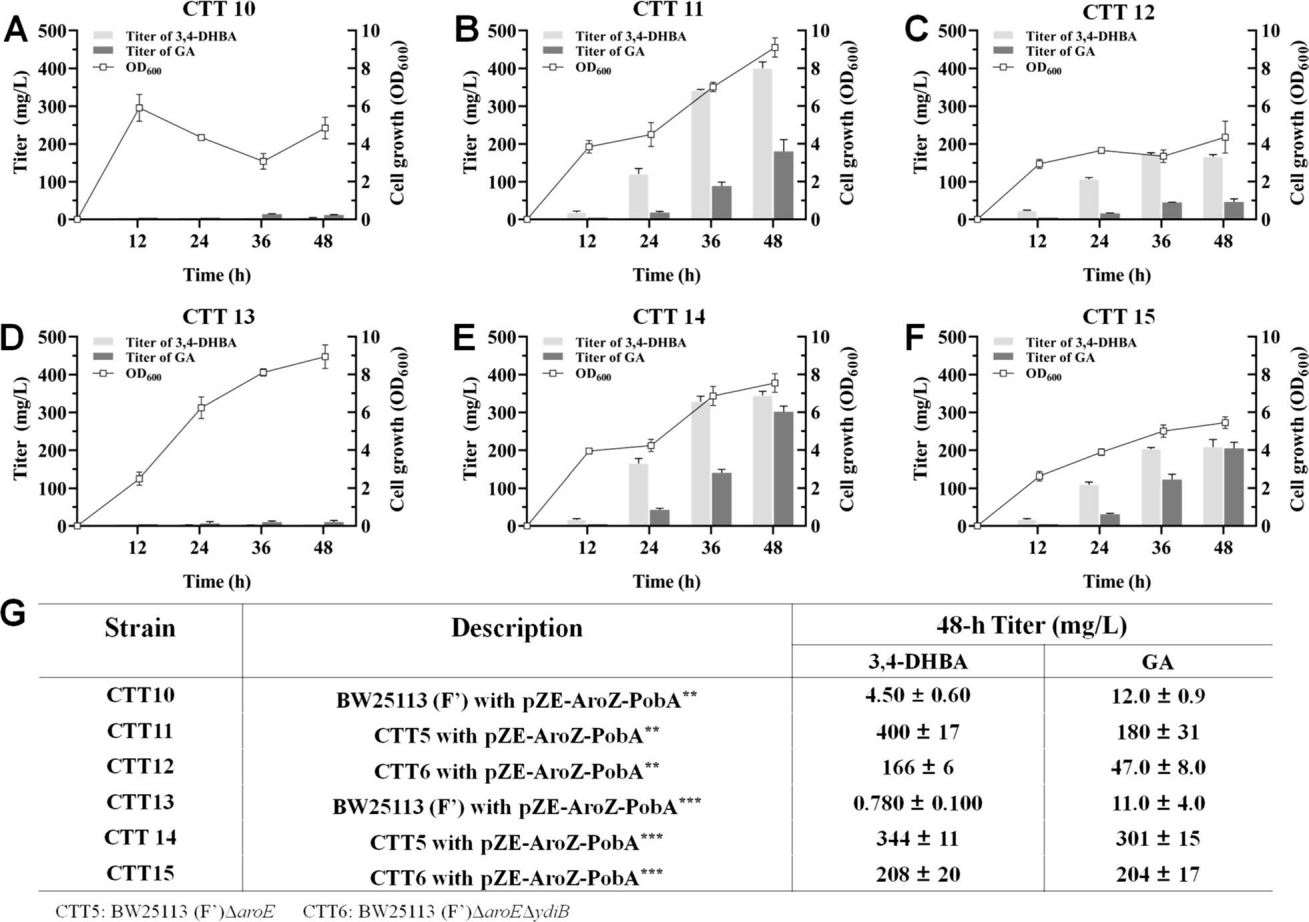
De novo production of GA. **A** Strain *E. coli* BW25113 (Fʹ) with pZE-AroZ-PobA^**^ (CTT10) was used. **B** Strain CTT5 with pZE-AroZ-PobA^**^ (CTT11) was used. **C** Strain CTT6 with pZE-AroZ-PobA^**^ (CTT12) was used. **D** Strain *E. coli* BW25113 (Fʹ) with pZE-AroZ-PobA^***^ (CTT13) was used. **E** Strain CTT5 with pZE-AroZ-PobA^***^ (CTT14) was used. **F** Strain CTT6 with pZE-AroZ-PobA^***^ (CTT15) was used. **G** The production of 3,4-DHBA and GA in 48 h with the strain CTT10, CTT11, CTT12, CTT13, CTT14 and CTT15

To achieve the de novo production of GA, plasmid pZE-AroZ-PobA^**^ was individually introduced into *E. coli* BW25113 (Fʹ), CTT5 and CTT6, generating strains CTT10, CTT11 and CTT12, respectively. The fermentation results are displayed in Fig. 6. For strain CTT10, the production of GA lasted up to 36 h. At 36 h, only 14.1 ± 1.0 mg/L GA accumulated in the culture (Fig. 6A). Besides, negligible 3,4-DHBA was observed in the culture, suggesting the generated 3,4-DHBA could be immediately converted into GA by strain CTT10. For strain CTT11, 3,4-DHBA and GA titers, as well as the cell growth, kept increasing throughout the 48-h fermentation (Fig. 6B). Significantly, within 48 h, 3,4-DHBA with a titer of 400 ± 17 mg/L and GA with a titer of 180 ± 31 mg/L were detected in the culture. Notably, CTT11 produced 12.8-fold higher amount of GA when compared with CTT10, indicating that knockout of *aroE* significantly increased the ability of *E. coli* BW25113 (Fʹ) to de novo produce GA. For strain CTT12, GA titer and OD_600_ value continued to increase during the 48-h fermentation (Fig. 6C). CTT12 has a GA titer of 46.5 ± 8.0 mg/L at 48 h. Significantly, GA titer of CTT12 was 3.87-fold lower than that of CTT11, suggesting that *E. coli* BW25113 (Fʹ)Δ*aroE*Δ*ydiB* has lower ability to de novo synthesize GA when compared with *E. coli* BW25113 (Fʹ)Δ*aroE*.

As a comparison, plasmid pZE-AroZ-PobA^***^ was individually introduced into *E. coli* BW25113 (Fʹ), CTT5 and CTT6, generating strains CTT13, CTT14 and CTT15, respectively. As shown in Fig. 6D, CTT13 produced trace amount of GA (10.9 ± 2.3 mg/L) as CTT10. For strain CTT14, the increasing trends of 3,4-DHBA and GA titers, and OD_600_ value were similar to that of CTT11 (Fig. 6E). Within 48 h, the accumulation of 3,4-DHBA reached 344 ± 11 mg/L, which were 1.16-fold lower than that of CTT11. Meanwhile, GA production reached 301 ± 15 mg/L, a 1.67-fold higher value when compared with that of CTT11, suggesting mutant Y385F/T294A/V349A PobA has stronger ability to de novo produce GA when compared with mutant Y385F/T294A PobA. Besides, results in Fig. 6F show strain CTT15 produced 208 ± 20 mg/L 3,4-DHBA and 204 ± 17 mg/L GA at 48 h, which were 1.25- and 4.39-fold higher than these of CTT12, respectively. Compared to that of CTT14, 3,4-DHBA and GA titers of CTT15 were 1.65- and 1.48-fold lower, respectively. Overall, introducing the designed artificial pathway into *E. coli* could achieve GA biosynthesis from simple carbon sources. *E. coli* BW25113 (Fʹ)Δ*aroE* demonstrates stronger ability for de novo producing GA when compared with *E. coli* BW25113 (Fʹ) or *E. coli* BW25113 (Fʹ)Δ*aroE*Δ*ydiB* (Fig. 6G). Assembling mutant Y385F/T294A/V349A PobA into GA biosynthetic pathway enabled more GA production than that of assembling Y385F/T294A PobA into GA biosynthetic pathway, which were consistent with the results of in vitro enzyme assay and in vivo conversion experiments.

### De novo production of pyrogallol

**Fig. 7:**
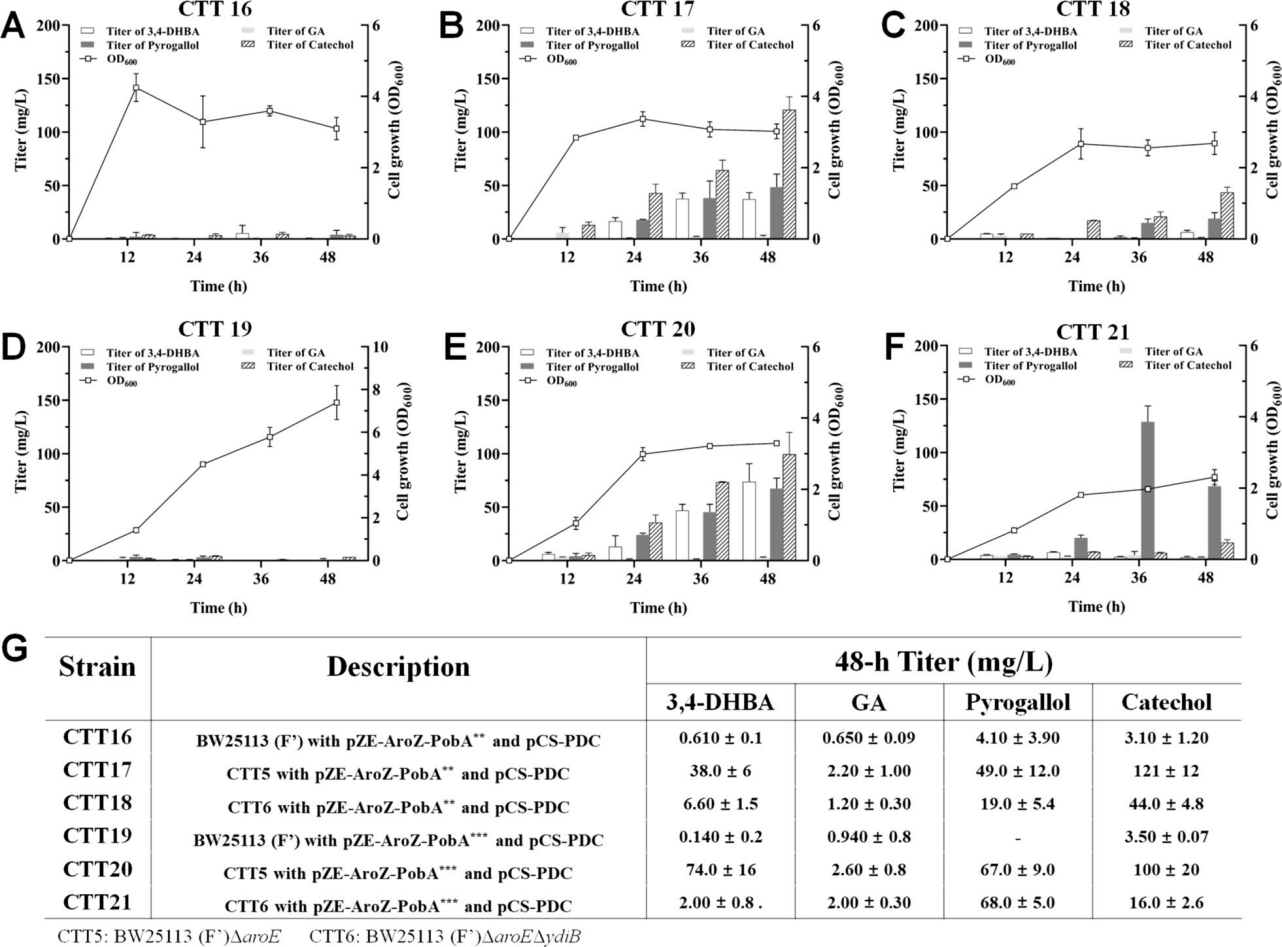
De novo production of pyrogallol. **A** Strain *E. coli* BW25113 (Fʹ) with pZE-AroZ-PobA^**^ and pCS-PDC (CTT16) was used. **B** Strain CTT5 with pZE-AroZ-PobA^**^ and pCS-PDC (CTT17) was used. **C** Strain CTT6 with pZE-AroZ-PobA^**^ and pCS-PDC (CTT18) was used. **D** Strain *E. coli* BW25113 (Fʹ) with pZE-AroZ-PobA^***^ and pCS-PDC (CTT19) was used. **E** Strain CTT5 with pZE-AroZ-PobA^***^ and pCS-PDC (CTT20) was used. **F** Strain CTT6 with pZE-AroZ-PobA^***^ and pCS-PDC (CTT21) was used. **G** The production of 3,4-DHBA, GA, catechol and pyrogallol with the strain CTT16, CTT17, CTT18, CTT19, CTT20 and CTT21 in 48 h

*E. coli* BW25113 (Fʹ) harboring pZE-AroZ-PobA^**^ and pCS-PDC (CTT16), CTT5 harboring pZE-AroZ-PobA^**^ and pCS-PDC (CTT17) and CTT6 harboring pZE-AroZ-PobA^**^ and pCS-PDC (CTT18) were constructed to de novo produce pyrogallol. The fermentation results of CTT16 showed trace amount of 3,4-DHBA, GA, pyrogallol and byproduct catechol accumulated in the culture throughout the 48-h fermentation (Fig. 7A). For CTT17, the OD_600_ value increased during the first 24 h and has no remarkable raise during the next 24 h (Fig. 7B). Pyrogallol and byproduct catechol titers increased throughout the 48-h fermentation, and reached 48.6 ± 12.0 and 121 ± 12 mg/L at 48 h, respectively. Besides, the accumulation of GA cannot be significantly detected in the culture, indicating that the generated GA was immediately converted into pyrogallol. For CTT18, pyrogallol and byproduct catechol titers raised continuously in 48 h, and reached 19.2 ± 5.4 and 43.7 ± 4.8 mg/L at 48 h, respectively (Fig. 7C). Significantly, pyrogallol and byproduct catechol titers were 2.53- and 2.77-fold lower than these of CTT17, which suggest *E. coli* BW25113 (Fʹ)Δ*aroE* performed better than *E. coli* BW25113 (Fʹ)Δ*aroE*Δ*ydiB* for de novo production of pyrogallol.

Subsequently, plasmids pZE-AroZ-PobA^***^ and pCS-PDC were co-transferred into *E. coli* BW25113 (Fʹ), CTT5 and CTT6, resulting in strains CTT19, CTT20 and CTT21, respectively. As shown Fig. 7D, CTT19 hardly produced 3,4-DHBA, GA, pyrogallol and catechol as CTT16. In Fig. 7E, CTT20 continued to grow in the first 24 h and stopped growing in the subsequent 24 h. CTT20 yielded 67.4 ± 9.7 mg/L pyrogallol at 48 h, which was 1.39-fold higher than that of CTT17. Meanwhile, 99.7 ± 20.3 mg/L catechol accumulated in the culture, which was 1.21-fold lower than that of CTT17. These indicate the efficiency of mutant Y385F/T294A/V349A PobA was higher than that of mutant Y385F/T294A PobA for de novo biosynthesis of pyrogallol. For CTT21, pyrogallol was continuously synthesized in the first 36 h (Fig. 7F) and has a titer of 129 ± 15 mg/L at 36 h, a 1.91-fold higher value when compared with that of CTT20. Meanwhile, only 6.12 ± 0.46 mg/L catechol were detected, which was 12.0-fold lower than that of CTT20 at 36 h. Within 48 h, pyrogallol titer decreased to 68.5 ± 5.0 mg/L and catechol increased to 15.8 ± 2.6 mg/L. These suggest CTT21 could achieve efficient de novo pyrogallol production, meanwhile, the accumulation of byproduct catechol was trace. In all, *E. coli* containing the designed artificial pathway could achieve the de novo biosynthesis of pyrogallol. Among the engineered strains, *E. coli* BW25113 (Fʹ)Δ*aroE*Δ*ydiB* with overexpression of Y385F/T294A/V349A PobA and PDC demonstrates strongest ability for de novo production of pyrogallol (Fig. 7G).

## Conclusion

The low hydroxylation activity of native PobA towards 3,4-DHBA limited the high-level production of GA and pyrogallol. Random mutagenesis was an efficient method to generate high activity PobA mutants. This work first established a simple screening method which based on the instability of GA under alkaline conditions. Using this screening method, a PobA mutant (Y385F/T294A/V349A PobA) with high activity towards 3,4-DHBA was screen out from a PobA random mutagenesis library. Y385F/T294A/V349A PobA possesses higher catalytic efficiency towards 4-HBA or 3,4-DHBA when compared with the reported mutant (Y385F/T294A PobA). Moreover, Y385F/T294A/V349A PobA represents higher in vivo ability of converting 3,4-DHBA into GA or pyrogallol when compared with Y385F/T294A PobA. Assembling Y385F/T294A/V349A PobA into the de novo GA or pyrogallol biosynthetic pathway achieved GA or pyrogallol production from simple carbon sources. In all, this work constructed an efficient method for screening high activity hydroxylase PobA, and this method could be applied for screening other GA biosynthesis-related enzymes. The high activity PobA which was obtained in this study has great potential for the production of GA or pyrogallol derivatives.

## Materials and methods

### Media, strains and plasmids

Luria–Bertani (LB) medium containing 10 g NaCl, 10 g tryptone and 5 g yeast extract per liter, was used for cell inoculation and propagation. For solid medium, 20 g/L agar was added. Modified M9 (M9Y) medium which contains 11.28 g/L 5 × M9 Minimal Salt, 10 g/L glycerol, 2.5 g/L glucose, 1 mM MgSO_4_, 0.05 mM CaCl_2_, 2 g/L MOPS and 5 g/L yeast extract, was used for feeding experiments and de novo production of GA and pyrogallol. Terrific Broth (TB) medium which contains 12 g/L tryptone, 24 g/L yeast extract, 4 g/L glycerol, 12.5 g/L K_2_HPO_4_ and 2.31 g/L KH_2_PO_4_, was used for protein expression. If needed, 100 μg/mL ampicillin or 50 μg/mL kanamycin was added to the culture. *E. coli* XL10-Gold and *E. coli* BL21(DE3) were used for plasmid construction and protein expression, respectively, while *E. coli* BW25113 (Fʹ) was used for feeding experiments and de novo biosynthesis of GA and pyrogallol. Plasmids pZE12-luc and pCS27 were used for pathway construction. Plasmid pETDuet-1 was used for protein expression. Plasmids pKD46 and pCP20 were used for knockout of genes. Strains and plasmids used in this study are depicted in Table 2 and Additional file 1: Table S1.

**Table 2:** Plasmids and strains used in this study

Plasmids and strains	Description	Source
Plasmids		
pZE12-luc	*P*_*L*_*lacO1; colE1; amp*^*r*^	Storage
pCS-PDC	*P*_*L*_*lacO1-PDC; P15A; kan*^*r*^	Storage
pETDuet-1	*P*_*T7*_*; pBR322; amp*^*r*^	Storage
pKD46	*P*_*araB*_*-*g*am-beta-exo; pCS101; amp*^*r*^	Storage
pCP20	*P*_*λpR*_-*flp; pCS101; amp*^*r*^*; cm*^*r*^	Storage
pZE-PobA^**^	*P*_*L*_*lacO1*-*Y385F/T294A PobA; colE1; amp*^*r*^	This study
pZE-PobA^***^	*P*_*L*_*lacO1*-*Y385F/T294A/V349A PobA; colE1; amp*^*r*^	This study
pETDuet-PobA^**^	*P*_*T7*_*-Y385F/T294A PobA; pBR322; amp*^*r*^	This study
pETDuet-PobA^***^	*P*_*T7*_*-Y385F/T294A/V349A PobA; pBR322; amp*^*r*^	This study
pZE-AroZ	*P*_*L*_*lacO1-AroZ; colE1; amp*^*r*^	This study
pZE-AroZ-PobA^**^	*P*_*L*_*lacO1*-*AroZ; P*_*L*_*lacO1-Y385F/T294A PobA; colE1; amp*^*r*^	This study
pZE-AroZ-PobA^***^	*P*_*L*_*lacO1*-*AroZ; P*_*L*_*lacO1-Y385F/T294A/V349A PobA; colE1; amp*^*r*^	This study
*E. coli* strains		
XL10-Gold	*TetrD(mcrA)183 D(mcrCB-hsdSMR-mrr)173 endA1 supE44 thi-1recA1 gyrA96 relA1 lac Hte [Fʹ proAB lacIqZ*Δ*M15 Tn10 (Tet*^*r*^*) Amy Cam*^*r*^*]*	Storage
BW25113 (Fʹ)	*rrnBT14* Δ*lacZWJ16 hsdR514* Δ*araBADAH33* Δ*rhaBADLD78 F*ʹ*[traD36 proAB lacI*^*q*^*Z*Δ*M15 Tn10(Tet*^*r*^*)]*	Storage
BL21(DE3)	*F*^*−*^*ompT hsdS (rB*^*−*^*mB*^*−*^*) gal dcm (DE3)*	Storage
CTT1	BW25113 (Fʹ) with pZE-PobA^**^	This study
CTT2	BW25113 (Fʹ) with pZE-PobA^***^	This study
CTT3	BW25113 (Fʹ) with pZE-PobA^**^ and pCS-PDC	This study
CTT4	BW25113 (Fʹ) with pZE-PobA^***^ and pCS-PDC	This study
CTT5	BW25113 (Fʹ)Δ*aroE*	This study
CTT6	BW25113 (Fʹ)Δ*aroE*Δ*ydiB*	This study
CTT7	BW25113 (Fʹ) with pZE-AroZ	This study
CTT8	CTT5 with pZE-AroZ	This study
CTT9	CTT6 with pZE-AroZ	This study
CTT10	BW25113 (Fʹ) with pZE-AroZ-PobA^**^	This study
CTT11	CTT5 with pZE-AroZ-PobA^**^	This study
CTT12	CTT6 with pZE-AroZ-PobA^**^	This study
CTT13	BW25113 (Fʹ) with pZE-AroZ-PobA^***^	This study
CTT14	CTT5 with pZE-AroZ-PobA^***^	This study
CTT15	CTT6 with pZE-AroZ-PobA^***^	This study
CTT16	BW25113 (Fʹ) with pZE-AroZ-PobA^**^ and pCS-PDC	This study
CTT17	CTT5 with pZE-AroZ-PobA^**^ and pCS-PDC	This study
CTT18	CTT6 with pZE-AroZ-PobA^**^ and pCS-PDC	This study
CTT19	BW25113 (Fʹ) with pZE-AroZ-PobA^***^ and pCS-PDC	This study
CTT20	CTT5 with pZE-AroZ-PobA^***^ and pCS-PDC	This study
CTT21	CTT6 with pZE-AroZ-PobA^***^ and pCS-PDC	This study

### DNA manipulation

Genes *Y385F/T294A pobA* [22] and *Y385F/T294A/V349A pobA* were individually cloned into pZE-luc by Gibson assembly, generating plasmids pZE-PobA^**^ and pZE-PobA^***^. Genes *Y385F/T294A pobA* and *Y385F/T294A/V349A pobA* were individually cloned into pETDuet-1, generating plasmids pETDuet-PobA^**^ and pETDuet-PobA^***^. To realize the efficient biosynthesis of 3,4-DHBA, gene *aroZ* (GenBank: VCW80955.1) was synthesized by OE-PCR, and was then inserted into pZE-luc by Gibson assembly, generating pZE-AroZ. In order to achieve GA production from simple carbon sources, *P*_*L*_*lacO1-AroZ* amplified from pZE-AroZ was individually inserted into pZE-PobA^**^ and pZE-PobA^***^ by Gibson assembly, resulting in plasmids pZE-AroZ-PobA^**^ and pZE-AroZ-PobA^***^. Plasmid pCS-PDC [23] was used for conversion of GA into pyrogallol. All the plasmids were confirmed through DNA sequencing.

### Establishment of a PobA mutagenesis library by error-prone PCR

To construct a *pobA* mutagenesis library, the in vivo activity and in vivo conversion ability of the reported PobA mutants (Y385F/T294A PobA [22], Y385F/L199V PobA [33], L199R/T294C/Y385M PobA [34] and V47I/L199N/T294A/Y385I PobA [34]) were compared. The in vitro activity is shown in Additional file 1: Table S2. The *k*_cat_/*K*_m_ value of L199R/T294C/Y385M PobA towards 3,4-DHBA was highest among the mutants. The *k*_cat_/*K*_m_ values of L199V/Y385F PobA and Y385F/T294A PobA were close to that of L199R/T294C/Y385M PobA. The in vivo conversion ability is shown in Additional file 1: Fig. S7. Y385F/T294A PobA exhibits highest in vivo conversion ability among the reported mutants. Taken in vitro and in vivo activities together, we chose *Y385F/T294A pobA* as the template for error-prone PCR to construct *pobA* random mutagenesis library because this work aimed to screen a high activity PobA mutant for achieving the efficient in vivo de novo production of GA in engineered *E. coli*. Plasmid pZE-pobA^**^ containing *Y385F/T294A pobA* was used as the template for error-prone PCR. First, 10× unbalanced dNTPs mixture which contained 2 mM dATP, 2 mM dGTP, 10 mM dCTP and 10 mM dTTP, was prepared. A 100 μL error-prone PCR reaction mixture included 1 μL 100 ng/μL pZE-pobA^**^, 10 μL 10× unbalanced dNTPs mixture, 0.5 μL 25 mM MnCl_2_, 1 μL 200 mM MgCl_2_, 1 μL 10 μM forward primer, 1 μL 10 μM reverse primer, 2 μL 5 U/μL Hieff™ Taq DNA Polymerase, 10 μL 10× M5 Taq PCR Buffer (Mg^2+^ free) and 73.5 μL ddH_2_O. The amplification program was as follows: 94 °C initial denaturation for 3 min, and then 30 cycles of 94 °C denaturation for 1 min, 58 °C annealing for 1 min and 72 °C extension for 1.5 min. After that, the PCR product was confirmed and purified via agarose gel electrophoresis. The purified product was digested by *Dpn*I at 37 °C for 1.5 h and then cloned into pZE-luc, generating a PobA mutagenesis library. This mutagenesis library contained 500 colonies.

### High-throughput screening of PobA mutants

The single colonies of PobA mutagenesis library were pre-inoculated into 96-deep-well plates which contained 1 mL LB and 100 μg/mL ampicillin, and then aerobically cultured at 37 °C for 12 h to generate seed cultures. After that, 10 μL seed cultures were transferred into 990 μL M9Y medium which was supplemented with 100 μg/mL ampicillin, 0.5 mM isopropyl-β-d-thiogalactopyranoside (IPTG) and 1 g/L 3,4-DHBA in 96-deep-well plates. The cultures were left at 30 °C for incubation. At the same time, the remaining seed cultures were pre-saved for DNA sequencing. After 12 h, samples were taken. The samples were firstly centrifuged at 12,000 rpm for 10 min to remove the cells and sediments in medium. After that, 50 μL supernatant was taken into 96-well plates which contained 0.1 M NaHCO_3_. After reaction for 2 h, the samples which with deepest green color among all the samples were screened out. Then, microplate reader (BioTek Cytation 3) was used to detect the optical densities of the screened samples at 640 nm. The mutations in the screened *pobAs* were confirmed through DNA sequencing.

### Expression and purification of PobA mutants

*E. coli* BL21 (DE3) containing pETDuet-PobA^**^ or pETDuet-PobA^***^ was pre-inoculated in 5 mL LB medium which contained 100 μg/mL ampicillin, and was then cultured overnight at 37 °C. Then, 1 mL of pre-inoculum was transferred into 100 mL of fresh TB containing 100 μg/mL ampicillin and cultured at 37 °C until OD_600_ reached around 0.6. After that, 0.5 mM IPTG was added to the culture to induce protein expression at 16 °C. After 12 h, the cells were harvested via centrifugation (4000 rpm for 30 min at 4 °C) and then resuspended in 20 mL lysis buffer (50 mM Tris–HCl, pH 7.5). The cells were disrupted by ultrasonic processor. The generated mixture was then centrifuged (10,000 rpm for 60 min at 4 °C). The supernatant was collected. The proteins were purified by nickel column. Quick Start™ Bradford Protein Assay (BIO-RED) was used for measuring protein concentrations.

### In vitro enzyme assay and modeling of PobA mutants

For HPLC detection, a 1000-μL reaction system which contained 100 mM Tris–HCl (pH 8.0), 10 μM FAD, 1000 μM NADPH, 500 nM purified enzyme and 0–1000 μM 4-HBA or 3,4-DHBA, was prepared. For NADPH detection, a 500-μL reaction system which contained 100 mM Tris–HCl (pH 8.0), 10 μM FAD, 1000 μM NADPH, 500 nM purified enzyme and 0–1000 μM 4-HBA or 3,4-DHBA, was prepared. The reactions were conducted at 30 °C for 2 min. The kinetic parameters were estimated through non-linear regression of the Michaelis–Menten equation in OriginPro8.5.

Structural models of Y385F/T294A PobA and Y385F/T294A/V349A PobA were built with the SWISS-MODEL server using Y385F PobA (PDB ID: 6JU1) as template [33]. The complexes of PobA mutants with 3,4-DHBA and FAD were modeled by AutoDock (version 4.2.6). The docking was performed in AutoDock (version 4.2.6) with the default settings, and the search box was resized to cover the reported binding pockets. After that, 50 docking models were obtained, and the model which was similar to the reported real structure (PDB ID: 6JU1) and with the lowest binding energy was considered as the correct model. In this study, the complex of Y385F/T294A PobA with 3,4-DHBA and FAD has a binding energy of − 5.99 kcal/mol and the complex of Y385F/T294A/V349A PobA with 3,4-DHBA and FAD has a binding energy of − 6.95 kcal/mol. The hydrogen bonds and other forces in the complexes were analyzed by PyMOL (version 2.4). The solvent accessible surface area (SASA) of the binding pocket in the complexes was calculated by PyMOL (version 2.4).

### Feeding experiments

To testify the in vivo conversion efficiency of PobA mutants towards 3,4-DHBA, feeding experiments were conducted. *E. coli* BW25113 (Fʹ) containing pZE-PobA^**^ (CTT1), *E. coli* BW25113 (Fʹ) containing pZE-PobA^***^ (CTT2), *E. coli* BW25113 (Fʹ) containing pZE-PobA^**^ and pCS-PDC (CTT3), and *E. coli* BW25113 (Fʹ) containing pZE-PobA^***^ and pCS-PDC(CTT4) were used. The single colonies were pre-inoculated into 5 mL LB with 100 μg/mL ampicillin and then cultured at 37 °C overnight. 200 μL overnight cultures were inoculated into 20 mL M9Y medium containing 100 μg/mL ampicillin. The cultures were cultivated at 37 °C for 2.5 h, and then induced with 0.5 mM IPTG at 30 °C. After induction for 3 h, 1000 mg/L 3,4-DHBA was fed to the culture. For GA production, samples were taken at 5.5, 6.5, 9, 12, 24, 36 and 48 h. For pyrogallol production, samples were taken at 12, 24, 36 and 48 h. Cell growth was confirmed through measuring OD_600_. Products and intermediates were analyzed by UHPLC.

### Knockout of genes *aroE* or *ydiB*

To acquire 3,4-DHBA-producing strains, gene *aroE* or *ydiB* of *E. coli* BW25113 (Fʹ) was knocked out. First, donor fragments for pre-knockout genes needed to be constructed. For knockout of gene *aroE*, 530 bp at the 5ʹ-terminus of *aroE,* FRT from pRecA-FRT [45], *kan*, FRT and 340 bp at the 3ʹ-terminus of *aroE* were sequentially assembled through OE-PCR, generating *aroE*-donor. For knockout of gene *ydiB*, *ydiB*-donor was constructed like *aroE*-donor. To construct *E. coli* BW25113 (Fʹ)Δ*aroE*, the *aroE*-donor fragment was transferred into *E. coli* BW25113 (Fʹ) containing pKD46, and then cultured at 30 °C overnight. To eliminate pKD46, the overnight cultures were then spread on LB solid medium with 50 μg/mL kanamycin and then cultured at 37 °C overnight. After that, plasmid pCP20 was transferred into the generated strain and cultured at 30 °C overnight. To induce FLP recombinase and eliminate plasmid pCP20, the single colonies were picked and individually incubated on LB solid medium with 100 μg/mL ampicillin, LB solid medium with 50 μg/mL kanamycin and LB solid medium overnight at 43 °C. The colonies which can grow on LB solid medium and cannot grow on LB solid medium with ampicillin or kanamycin, were the colonies of strain *E. coli* BW25113 (Fʹ)Δ*aroE* (CTT5). The construction of *E. coli* BW25113 (Fʹ)Δ*aroE*Δ*ydiB* (CTT6) was similar to that of CTT5.

### De novo production of 3,4-DHBA, GA and pyrogallol

*E. coli* BW25113 (Fʹ) containing pZE-AroZ (CTT7), CTT5 containing pZE-AroZ (CTT8) and CTT6 containing pZE-AroZ (CTT9), were used for de novo biosynthesis of 3,4-DHBA. *E. coli* BW25113 (Fʹ) containing pZE-AroZ-PobA^**^ (CTT10), CTT5 containing pZE-AroZ-PobA^**^ (CTT11), CTT6 containing pZE-AroZ-PobA^**^ (CTT12), *E. coli* BW25113 (Fʹ) containing pZE-AroZ-PobA^***^ (CTT13), CTT5 containing pZE-AroZ-PobA^***^ (CTT14) and CTT6 containing pZE-AroZ-PobA^***^ (CTT15), were used for de novo biosynthesis of GA. *E. coli* BW25113 (Fʹ) containing pZE-AroZ-PobA^**^ and pCS-PDC (CTT16), CTT5 containing pZE-AroZ-PobA^**^ and pCS-PDC (CTT17), CTT6 containing pZE-AroZ-PobA^**^ and pCS-PDC (CTT18), *E. coli* BW25113 (Fʹ) containing pZE-AroZ-PobA^***^ and pCS-PDC (CTT19), CTT5 containing pZE-AroZ-PobA^***^ and pCS-PDC (CTT20) and CTT6 containing pZE-AroZ-PobA^***^ and pCS-PDC (CTT21), were used for de novo biosynthesis of pyrogallol. Transformants were pre-inoculated into 5 mL LB medium with appropriated antibiotics and cultured overnight at 37 °C. Then, 200 μL seed cultures were transferred into 20 mL M9Y medium containing appropriated antibiotics and 0.5 mM IPTG. The cultures were cultivated at 30 °C for 48 h. Samples were collected at 12, 24, 36 and 48 h. OD_600_ values were measured. UHPLC was used for analyzing the products and intermediates.

### UHPLC analysis

The standards (3,4-DHBA, GA, catechol and pyrogallol) were purchased from J&K Chemicals. UHPLC (Agilent Technologies 1290 Infinity II), equipped with a reverse phase column (Agilent ZORBAX SB-C18, 5 μm, 4.6 × 250 mm), was used for analyzing and quantifying standards and samples. Firstly, the samples were centrifuged at 12,000 rpm for 10 min to remove the cells and sediments in medium. Then, the supernatants were filtered by 0.22 μm membrane and loaded. The column temperature was set at 30 °C. Flowing phase containing solvent A (water with 0.1% formic acid) and solvent B (100% methanol) were used, with a flow rate of 1 mL/min. The gradients were used as follows: 5% to 40% solvent B for 20 min, 100% solvent B for 2 min, 100% to 5% solvent B for 2 min and 5% solvent B for an additional 6 min. 3,4-DHBA, GA, catechol and pyrogallol were quantified based on their peak areas at 268 nm.

## Supplementary Information

**Additional file 1: Figure S1.** Analyzing the mixture of GA and NaHCO_3_ by UHPLC. **Figure S2.** MS analysis of the reaction mixture and speculation of compound in the reaction mixture. **Figure S3.** SDS-PAGE of Y385F/T294A PobA and Y385F/T294A/V349A PobA. **Figure S4.** The non-linear regression curves of PobA mutants towards 4-HBA and 3,4-DHBA through the Michaelis–Menten equation.** Figure S5.** Close view of the catalytic pocket of Y385F PobA with FAD and 3,4-DHBA complex. The hydrogen bonds were shown as dashed line. **Figure S6.** Verification of *E. coli* BW25113 (Fʹ), *E. coli* BW25113 (Fʹ)Δ*aroE* (CTT5) and *E. coli* BW25113 (Fʹ)Δ*aroE*Δ*ydiB* (CTT6). **Figure S7.** In vivo conversion of 3,4-DHBA into GA. **Table S1.** Plasmids and strains used in this study. **Table S2.** Kinetic parameters of PobA mutants towards 4-HBA and 3,4-DHBA.

## Data Availability

All data generated or analyzed during this study are included in this published article and its additional files.
