# Supplementary material for: A high-throughput visual screening method for p-hydroxybenzoate hydroxylase to increase phenolic compounds biosynthesis
Source: Biotechnol Biofuels Bioprod. 2022 May 2;15:43. doi: 10.1186/s13068-022-02142-w (PMC9063093; doi:10.1186/s13068-022-02142-w)
Supplement: Supplementary file 1 — Additional file 1: Figure S1. Analyzing the mixture of GA and NaHCO3 by UHPLC. Figure S2. MS analysis of the reaction mixture and speculation of compound in the reaction mixture. Figure S3. SDS-PAGE of Y385F/T294A PobA and Y385F/T294A/V349A PobA. Figure S4. The non-linear regression curves of PobA mutants towards 4-HBA and 3,4-DHBA through the Michaelis–Menten equation. Figure S5. Close view of the catalytic pocket of Y385F PobA with FAD and 3,4-DHBA complex. The hydrogen bonds were shown as dashed line. Figure S6. Verification of E. coli BW25113 (Fʹ), E. coli BW25113 (Fʹ)ΔaroE (CTT5) and E. coli BW25113 (Fʹ)ΔaroEΔydiB (CTT6). Figure S7. In vivo conversion of 3,4-DHBA into GA. Table S1. Plasmids and strains used in this study. Table S2. Kinetic parameters of PobA mutants towards 4-HBA and 3,4-DHBA. [file 13068_2022_2142_MOESM1_ESM.docx]

**A high-throughput visual screening method for *p*-hydroxybenzoate hydroxylase to increase phenolic compounds biosynthesis**

Zhenya Chen, Tongtong Chen, Shengzhu Yu, Yi-Xin Huo^*^

**Additional Information**

**Contents:**

Fig. S1

Fig. S2

Fig. S3

Fig. S4

Fig. S5

Fig. S6

Fig. S7

Table S1

Table S2


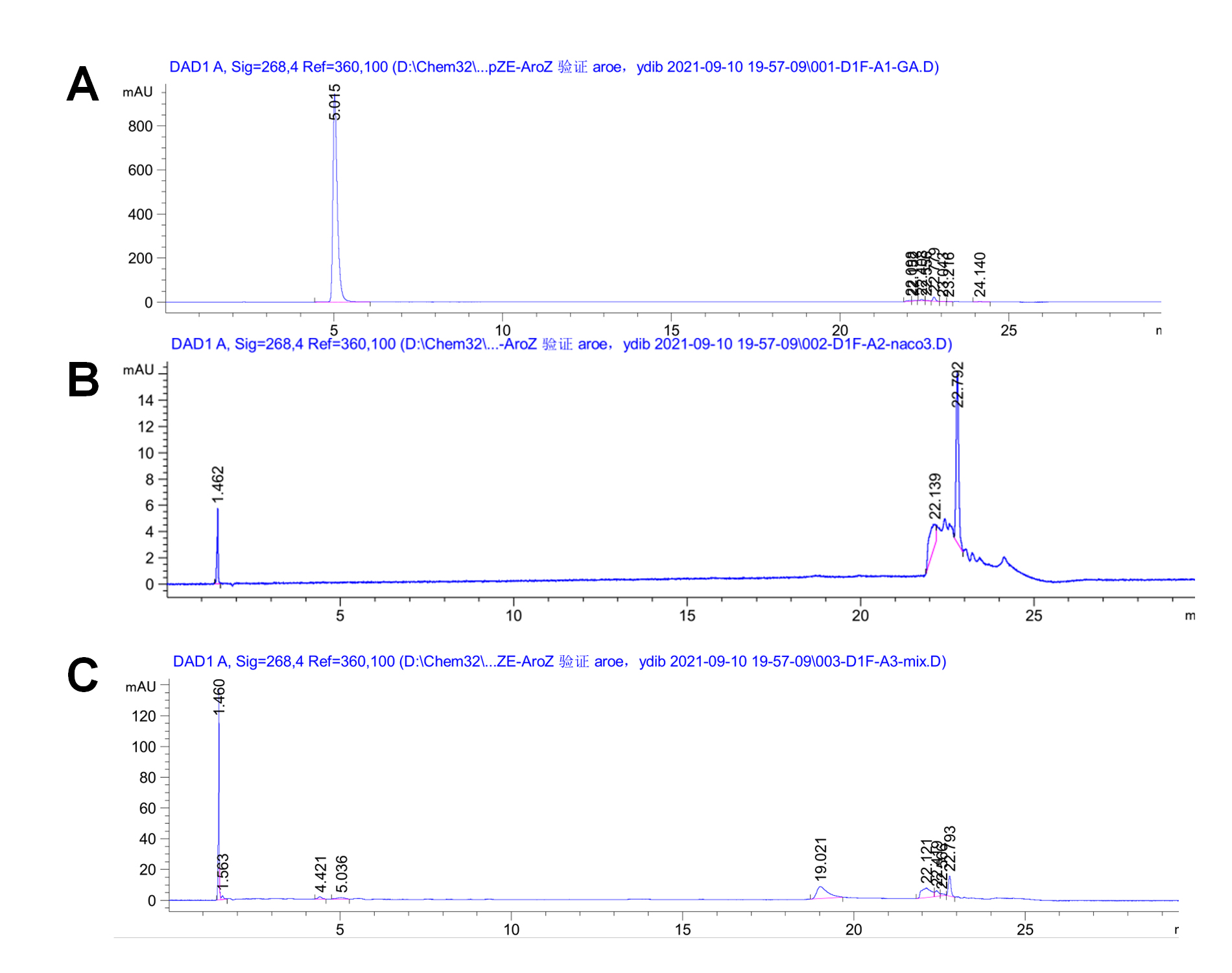


**GA**

**Fig. S1** Analyzing the mixture of GA and NaHCO_3_ by UHPLC. **(A)** UHPLC graph of 200 mg/L GA. **(B)** UHPLC graph of 0.1 M NaHCO_3_. **(C)** UHPLC graph of the reaction mixture.


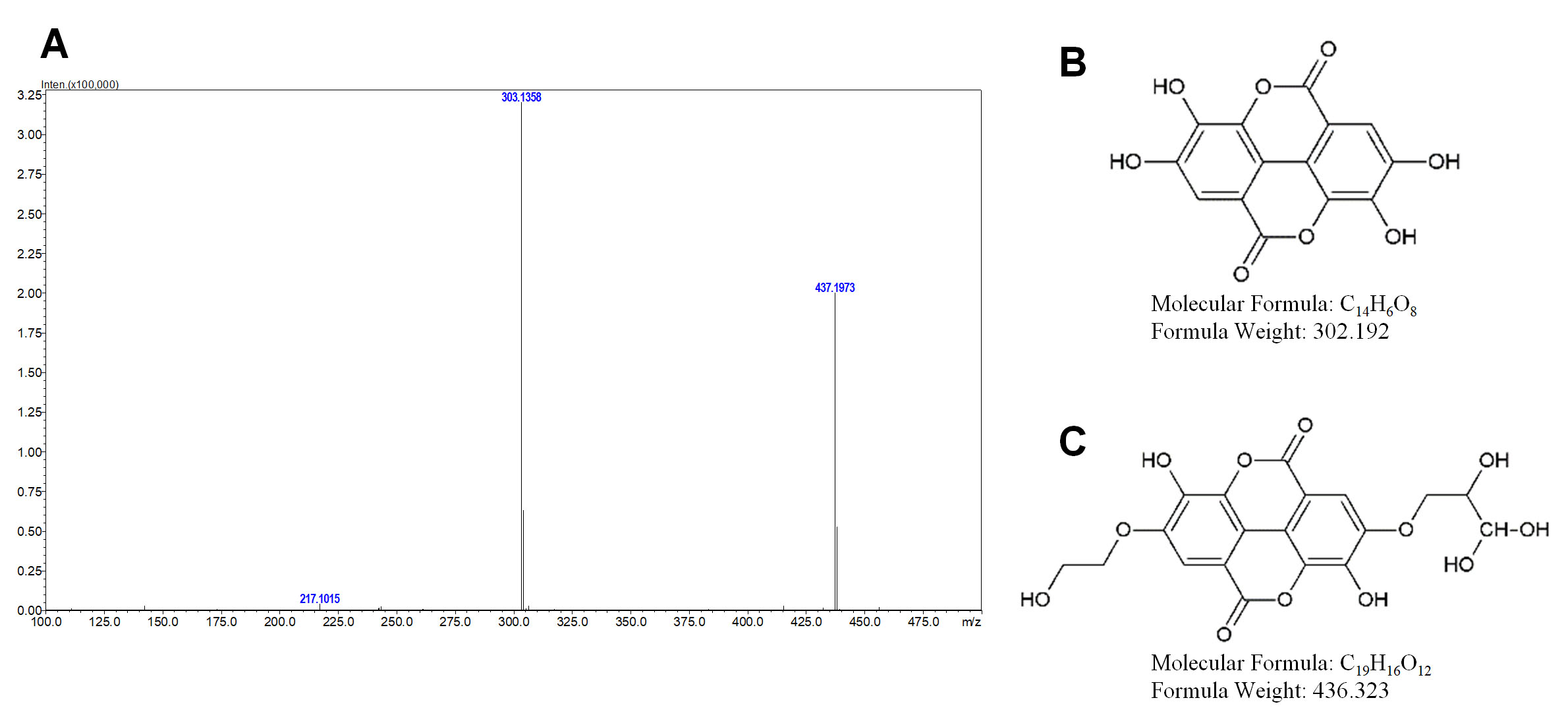


**Fig. S2** MS analysis of the reaction mixture and speculation of compound in the reaction mixture. **(A)** MS graph of the reaction mixture. **(B)** The speculative structure of the new compound (FW: 302.192). **(C)** The speculative structure of the new compound (FW: 436.323).


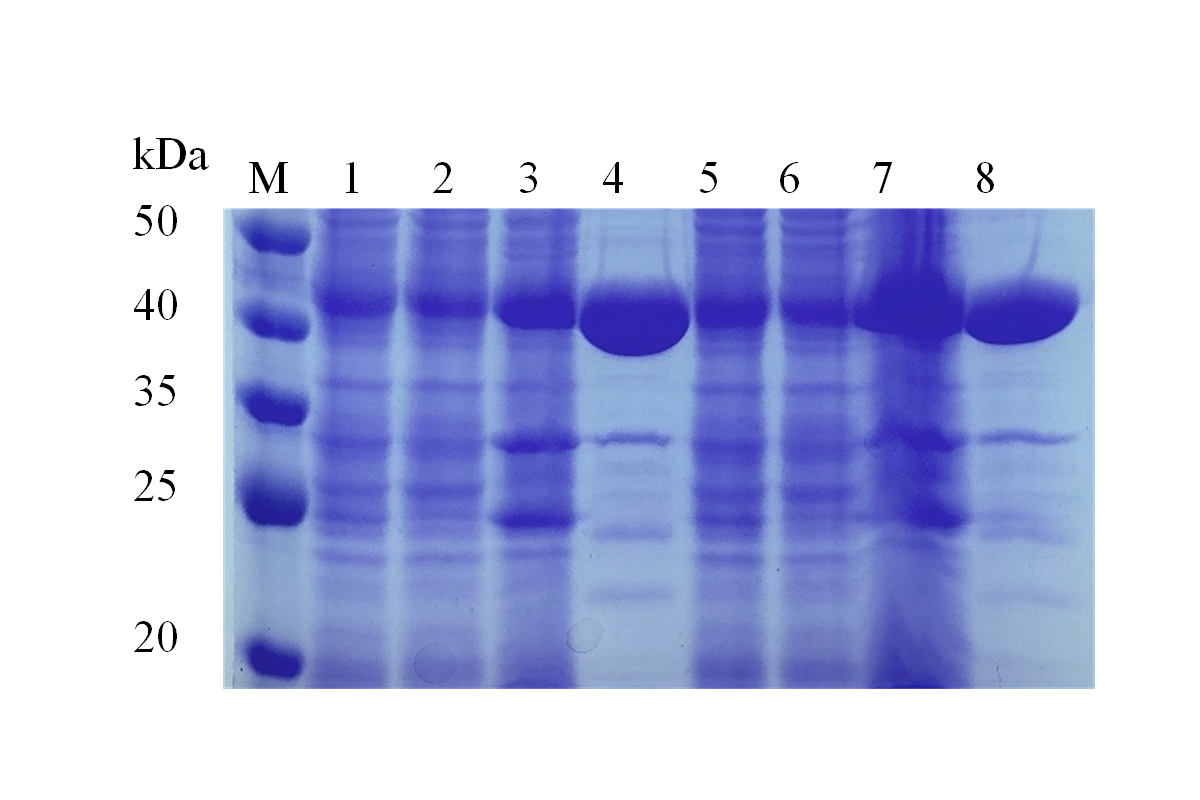


**Fig. S3** SDS-PAGE of Y385F/T294A PobA and Y385F/T294A/V349A PobA. Lane M: 50 kDa protein molecular weight marker. Lane 1: the lysed mixture after ultrasonic treatment of Y385F/T294A PobA. Lane 2 and 3: the precipitate and the supernatant of the lysed mixture of Y385F/T294A PobA after centrifugation, respectively. Lane 4: the purified Y385F/T294A PobA. Lane 5: the lysed mixture after ultrasonic treatment of Y385F/T294A/V349A PobA. Lane 6 and 7: the precipitate and the supernatant of the lysed mixture of Y385F/T294A/V349A PobA after centrifugation, respectively. Lane 8: the purified Y385F/T294A/V349A PobA.


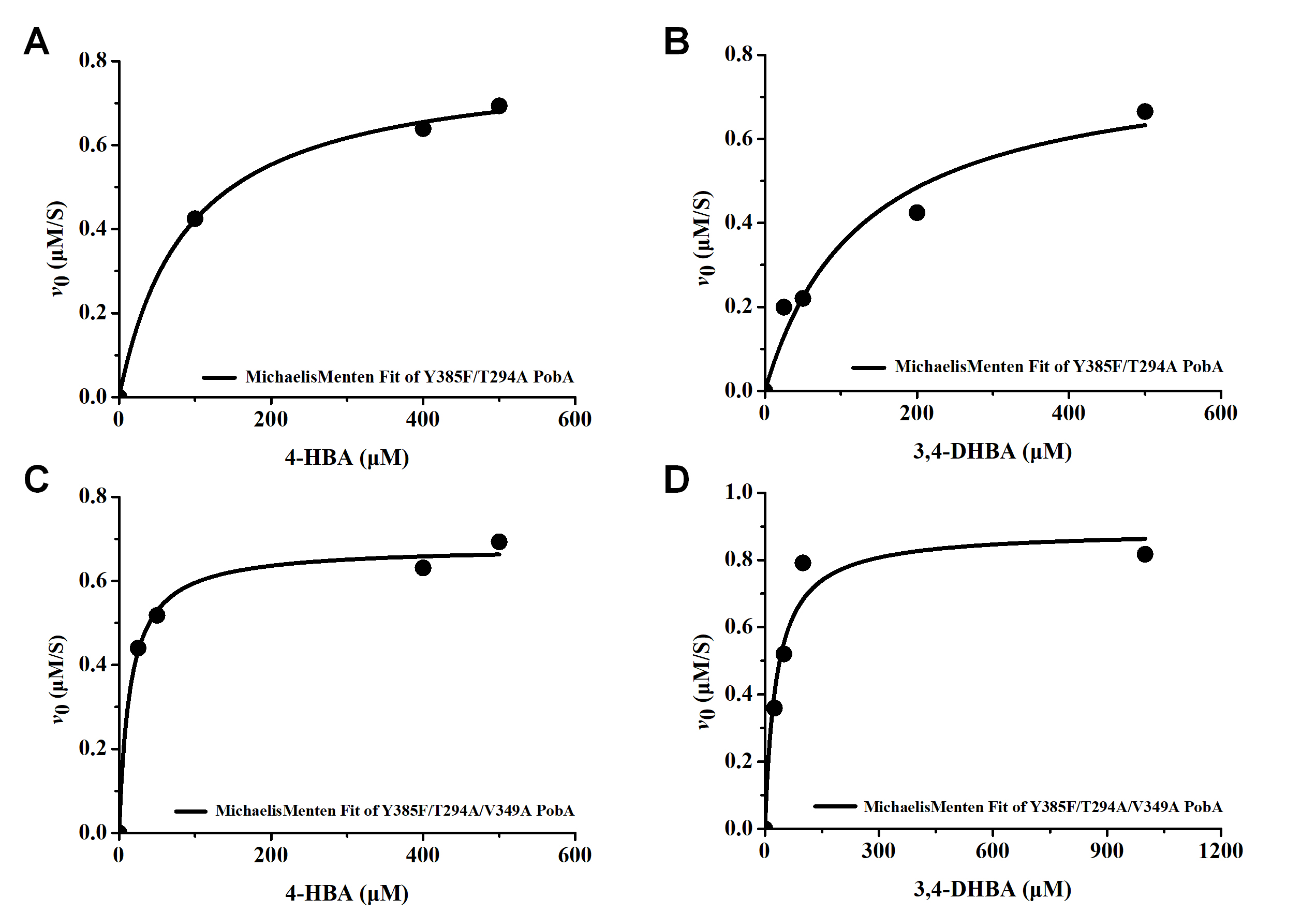


**Fig. S4** The non-linear regression curves of PobA mutants towards 4-HBA and 3,4-DHBA through the Michaelis-Menten equation. **(A)** The non-linear regression curves of Y385F/T294A PobA towards 4-HBA. **(B)** The non-linear regression curves of Y385F/T294A PobA towards 3,4-DHBA. **(C)** The non-linear regression curves of Y385F/T294A/V349A PobA towards 4-HBA. **(D)** The non-linear regression curves of Y385F/T294A/V349A PobA towards 3,4-DHBA.

**
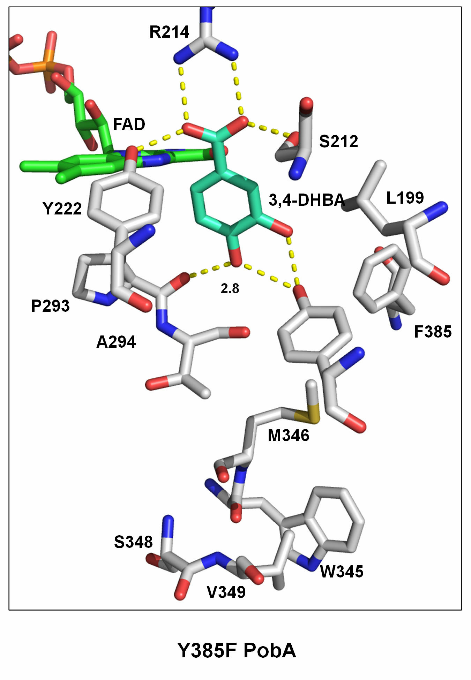
**

**Fig. S5** Close view of the catalytic pocket of Y385F PobA with FAD and 3,4-DHBA complex. The hydrogen bonds were shown as dashed line.


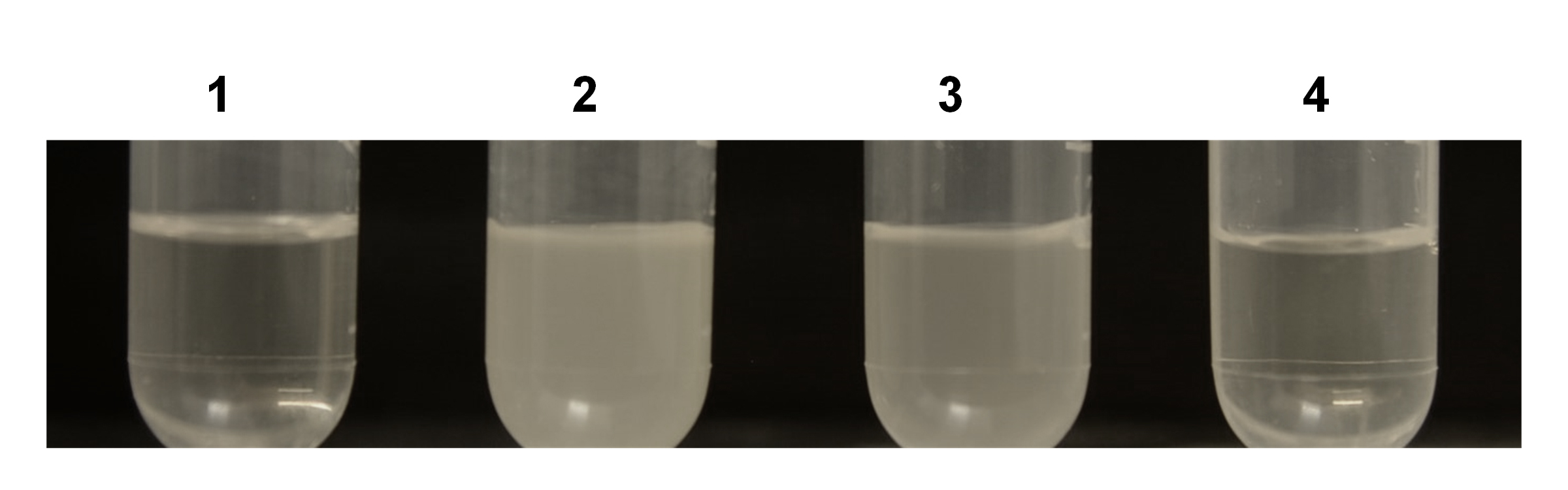


**Fig. S6** Verification of *E. coli* BW25113 (F’), *E. coli* BW25113 (F’)Δ*aroE* (CTT5) and *E. coli* BW25113 (F’)Δ*aroE*Δ*ydiB* (CTT6). 1: Only M9Y medium without Yeast Extract. 2: *E. coli* BW25113 (F’) in M9Y medium without Yeast Extract. 3: CTT5 with M9Y medium without Yeast Extract. 4: CTT6 in M9Y medium without Yeast Extract.


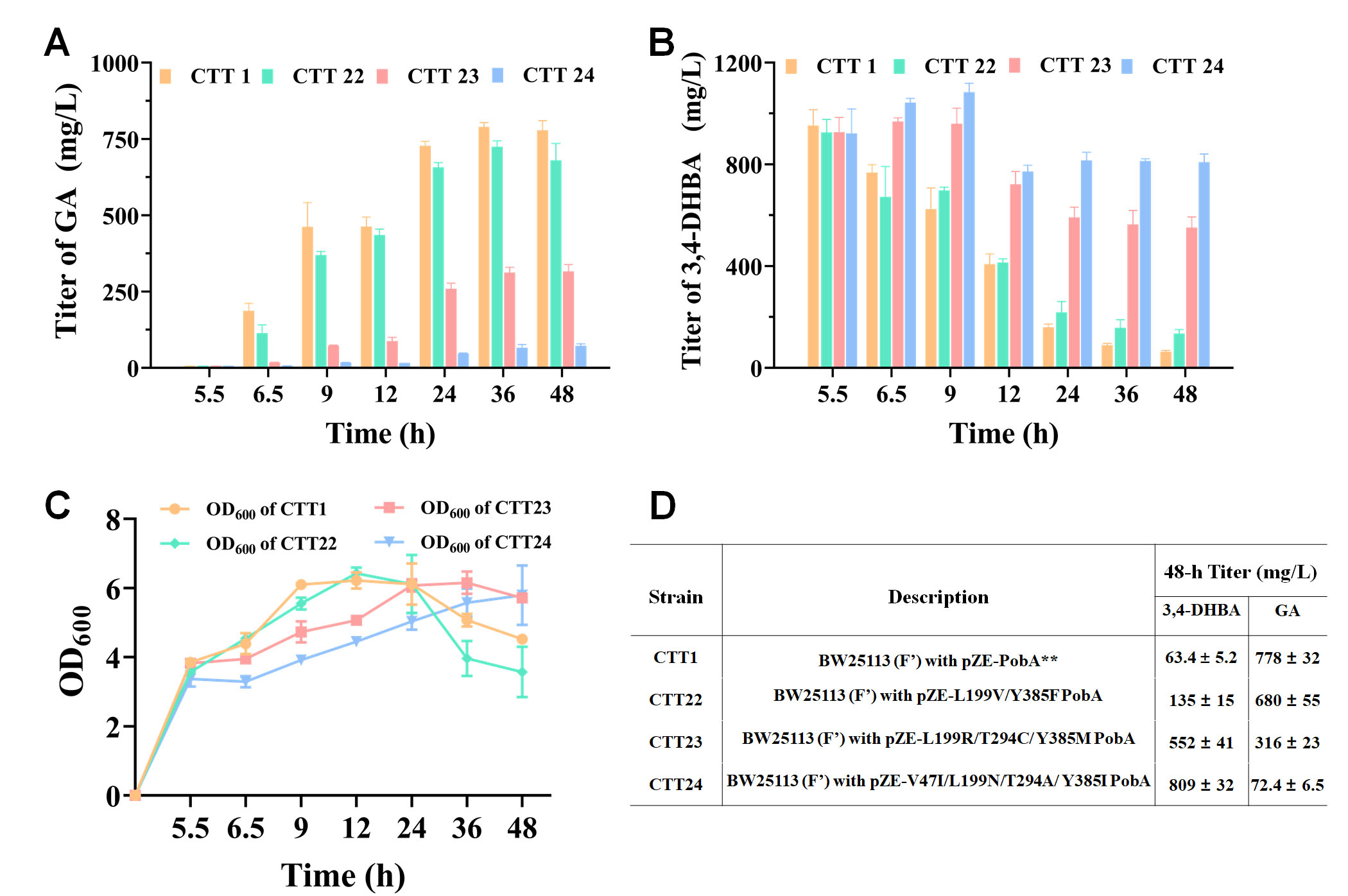


**Fig. S7** *In vivo* conversion of 3,4-DHBA into GA. 3,4-DHBA with a concentration of 1000 mg/L was fed to the culture at 5.5 h. **(A)** The titer of GA. **(B)** The titer of 3,4-DHBA. **(C)** The OD_600_ of strain CTT1, CTT22, CTT23 and CTT24. **(D)** The production of GA in 48 h with the strain with the strain CTT1, CTT22, CTT23 and CTT24.

**Table S1** Plasmids and strains used in this study

| Plasmids and strains | Description | Source |
| --- | --- | --- |
| Plasmids |  |  |
| pZE-Y385F/L199V PobA | *P_L_lacO1*-*Y385F/L199V PobA; colE1; amp^r^* | This study |
| pZE-L199R/T294C/Y385M PobA | *P_L_lacO1*-*L199R/T294C/Y385M PobA; colE1; amp^r^* | This study |
| pZE-V47I/L199N/T294A/Y385I PobA | *P_L_lacO1*-*V47I/L199N/T294A/Y385I PobA; colE1; amp^r^* | This study |
| *E. coli* strains |  |  |
| BW25113 (F’) | *rrnBT14* Δ*lacZWJ16 hsdR514* Δ*araBADAH33* Δ*rhaBADLD78 F’[traD36 proAB lacI^q^Z*Δ*M15 Tn10(Tet^r^)]* | Storage |
| CTT22 | BW25113 (F’) with pZE-Y385F/L199V PobA | This study |
| CTT23 | BW25113 (F’) with pZE-L199R/T294C/Y385M PobA | This study |
| CTT24 | BW25113 (F’) with pZE-V47I/L199N/T294A/Y385I PobA | This study |

**Table S2 Kinetic parameters of PobA mutants towards 4-HBA and 3,4-DHBA**

| PobA mutants | 4-HBA | | | | | | 3,4-DHBA | | | | | |
| --- | --- | --- | --- | --- | --- | --- | --- | --- | --- | --- | --- | --- |
|  | *K*_m_  (μM) | | *k*_cat_  (s^-1^) | | *k*_cat_/*K*_m_  (μM^-1.^s^-1^) | | *K*_m_  (μM) | | *k*_cat_  (s^-1^) | | *k*_cat_/*K*_m_  (μM^-1.^s^-1^) | |
| Y385F/T294A | 89.9 ± 11.2 | 1.60 ± 0.05 | | 0.018 | | 128 ± 52 | | 1.59 ± 0.32 | | 0.012 | |  |
| L199V/Y385F | 45.9 ± 43.0 | 0.744 ± 0.220 | | 0.0162 | | 84.9 ± 10.4 | | 1.17 ± 0.72 | | 0.0138 | |  |
| L199R/T294C/Y385M | 18.8 ± 7.8 | 0.699 ± 0.050 | | 0.0538 | | 40.3 ± 42.7 | | 0.678 ± 0.208 | | 0.0168 | |  |
| V47I/L199N/T294A / Y385I | 291 ± 42 | 1.65 ± 0.11 | | 0.0566 | | 448 ± 143 | | 3.23 ± 0.55 | | 0.00721 | |  |
